# Construction of the metabolism-related models for predicting prognosis and infiltrating immune phenotype in lung squamous cell carcinoma

**DOI:** 10.7150/jca.86942

**Published:** 2023-10-24

**Authors:** Zeming Ma, Liang Wang, Xiaoyun Huang, Hong Ji, Haoyang Wang, Yue Yang, Yuanyuan Ma, Jinfeng Chen

**Affiliations:** 1 Department of Thoracic Surgery II, Key Laboratory of Carcinogenesis and Translational Research (Ministry of Education), Peking University Cancer Hospital and Institute, Beijing, 100142, People's Republic of China.; 2 Department of Computational Oncology, Intelliphecy, Shenzhen, China; Center for Systems Biology, Intelliphecy, Shenzhen, China.; 3 Department of Gastrointestinal Oncology, Key Laboratory of Carcinogenesis and Translational Research (Ministry of Education/Beijing), Peking University Cancer Hospital and Institute, 100142, People's Republic of China.; 4 Beijing International Bilingual Academy, People's Republic of China.

**Keywords:** metabolism, prognosis, immune phenotype, lung squamous cell carcinoma, STXBP1

## Abstract

**Purpose:** Cancers often display disorder metabolism, which closely related to the poor outcome of patients. We aimed to establish prognostic models using metabolism-associated genes, and identify the key factor involved in metabolism in lung squamous cell carcinoma (LUSC).

**Materials and Methods:** R package 'TCGA biolinks' was used to download the mRNA sequencing data of LUSC from TCGA. The clusterProfiler package was performed to analyze biological pathways. The online tool GEPIA2 and cox regression method were applied to identify the two gene lists associated with metabolism and prognosis of LUSC. The lasso modeling was conducted to establish prognostic models. The quantiseq method was used to identify the cellular abundance of expression matrix in TCGA-LUSC dataset. Immunohistochemistry and western blotting were done to evaluate the STXBP1 expression in LUSC samples. Lactate assay and ATP detection were performed to assess metabolic effect, and CCK8 assay was done to test cell proliferation in the LUSC cells with overexpression and suppression of STXBP1.

**Results:** Two lists of survival-metabolism-associated genes (11 and 28 genes) were identified and applied in the prognostic model 1 and model 2 construction from TCGA-LUSC dataset. High-risk LUSC patients associated with poor survival in the training cohort and the test cohort of both model 1 and model 2. Higher ROC values for 10- year survival was shown in model 2 than in model 1. In addition, macrophage M1, macrophage M2, neutrophil, and T regulatory cell were enriched in the high-risk group of model 2. STXBP1 was the only optimized gene in both model 1 and model 2, and related to the poor outcome of LUSC patients. Furthermore, STXBP1 associated with infiltrating immune cells, and increased lactate, ATP levels, and cell proliferation.

**Conclusion:** Our finding provides the metabolism-associated models to predict prognosis of LUSC patients. STXBP1, as the key optimized gene in the model, promotes metabolic progress to increase lactate and ATP levels in LUSC cells.

## Introduction

Lung cancer has become one of leading cause of cancer-related death worldwide 1. Almost 85% of lung cancer is non-small cell lung cancer (NSCLC) including the major types of lung adenocarcinoma (LUAD) and lung squamous cell carcinoma (LUSC) 2, 3. As the mostly common targeted therapy, EGFR tyrosine kinase inhibitors (EGFR-TKIs) have mainly showed effective therapy for the LUAD patients with activating EGFR mutations 4-6. However, the patients with LUSC are lack of the effective targets and show poor prognosis, compared to the LUAD patients. The essential targets for progression and development of LUSC are required.

Metabolic reprogramming has been known as a hallmark in several cancer types including NSCLC 7, 8. Compared to normal cells, cancer cell metabolism (CCM) shows specific properties and altered signaling, which associate with proliferation, progression and metastasis 9. There are increasing studies on glycolysis metabolism in the tumor environment, of which cancer cells undergo glycolysis and produce lactate to provide ATP 10-12. In addition, lactate is considered as byproduct under glycolysis response, which can influence the surrounding tumor cells and the infiltrating immune cells 13, 14.

In current study, we aimed to establish the prognostic model associated with CCM in LUSC. Based on lasso modeling, the CCM-prognostic models were constructed. In addition, the relation between optimal model and infiltrating immune cells was analyzed. Furthermore, we identified biological function for the important gene in the CCM-prognostic models.

## Materials and Methods

### Data collection and clinical data

The data of mRNA expression for LUSC was downloaded from the TCGA database including 501 cases (493 cases eligible for survival analysis) using R package 'TCGA biolinks'. The downloaded data from LUSC patients were included, and then the cases without overall survival (OS) information were excluded. The CCM genes were obtained from the CCM Gene DataBase (https://bioinfo.uth.edu/ccmGDB/) 15. Kaplan-Meier survival analyses of CCM genes were obtained from GEPIA 2 (Gepia2.cancer-pku.cn).

117 cases of tumor tissues were obtained the LUSC patients who received surgical resection between 2010 to 2014 from Peking university cancer hospital. The cases with pathological evaluation of LUSC were included, and then the cases without information including disease free survival (DFS) and OS were excluded.

### Pathway enrichment

Pathway enrichment analysis was performed using the clusterProfiler package to identify overrepresented biological pathways. Gene ontology (GO) analysis was conducted to identify enriched biological processes (BP) with a p-value cutoff of 0.01 and a q-value cutoff of 0.05. KEGG analysis was conducted with a p-value cutoff of 0.01 and a q-value cutoff of 0.05. To correct for multiple comparisons, we used the "BH" method for both GO and KEGG enrichment.

### Identification of survival metabolic genes

We constructed two lists of survival genes. The first list was derived from the most differential survival genes (DSG) provided by the online tool GEPIA2 (Gepia2.cancer-pku.cn). Alternatively, the second list was obtained by Cox regression. Median expression value was used to stratify patients into high expression group and low expression group. Uni-variate cox regression was performed for each gene. The hazard ratio and p-value for each gene were derived. Genes with p-value smaller than 0.05 were considered as median derived survival genes (MDSG). The most differential survival genes have the most significant association with patient overall survival. CCM associated gene list was obtained from the previous study 15. The overlap gene list between MDSG and CCM, as well as the overlap gene list between DSG and CCM were determined.

### Lasso Modeling

To construct predictive models with lasso modeling, the survival metabolic genes were used as model input. The number of metabolic genes is 11 for DSG and 28 for MDSG. Predictive model was then constructed on the most frequent geneset with effective coefficients in the lasso regression using the R package 'glmnet' for 1000 iterations on the training dataset, which consisted of 70% of the patients. The risk score was calculated as the sum of the normalized expression of genes multiplied by their coefficients in the geneset. The receiver operating characteristic (ROC) curve was utilized to determine the cutoff of risk scores as a predictor of three, five, and ten-year survival in LUSC patients prior to death. Patients were divided into two groups based on their risk scores, and survival analysis was conducted using 'Survminer' for both the training and testing datasets.

### Inference of immune infiltration

To estimate the cellular composition of the TCGA-LUSC dataset, deconvolution of cellular composition was performed using “immunedeconv” package. The quantiseq method was employed to infer the cellular abundance using the expression matrix of TCGA-LUSC dataset. The absolute fractions of ten immune cell subtypes were quantified from bulk RNA-seq data. The risk score calculated by lasso model was used to stratify patients into high risk and low risk groups. The statistical significance of differential immune cell subtype percentage was tested using “wilcox” method.

### Immunohistochemistry and Kaplan-Meier survival analysis

The immunohistochemistry was done on the paraffin-embedded sections from tumor tissues of LUSC patients in Peking University Cancer Hospital (n=117). The paraffin-embedded sections were dewaxed and hydrated by xylene and gradient alcohol. The endogenous peroxidase enzymes were blocked with H_2_O_2_. STXBP1 (Proteintech, 11459-1-AP) antibody was stained on the sections at 4 ℃ overnight. HRP-conjugated secondary antibody (ZSGB-BIO, PV-6000) was added on the sections at room temperature for 30 minutes. Then, DAB kit (ZSGB-BIO, ZLI-9017) was used to visualize the brown color. The sections were analyzed by the pathologists independently. Based on the evaluation of immunohistochemistry variables in our previous study 16 and *Allred et al.* 'paper 17, 0-1 and >1 were considered low expression and high expression, respectively.

The χ^2^ test and Fisher's exact test were used to analyze the correlation between the expression of STXBP1 and patients' clinical variables. The Kaplan-Meier method and the log-rank test were used to examine the DFS and OS of LUSC patients.

### Construction of STXBP1 network and infiltrated immune cells

The STXBP1 network was built with GeneMANIA (http://genemania.org). The infiltrated immune cells of LUSC samples with STXBP1 was analyzed by TIMER (https://cistrome.shinyapps.io/timer/).

### Cell culture and cell transfection

The human LUSC cell lines H1703, H2170, H226, and H520, and normal lung epithelial cell line BEAS-2B were cultivated in the 1640 medium with 10% FBS at 37 ˚C with 5% CO_2_. The lentivirus with ectopic STBXP1 was infected with the LUSC cells. The shRNAs of STBXP1 (shRNA1-STXBP1: GGACTCCGATTATCAAGGA, shRNA2-STXBP1: CAAGCTCGATGCCTATAAA, shRNA3-STXBP1: GGACAAACTTGACACCAAA) were transfected into the cells.

### Western blotting

The protein was lysed with RIPA buffer (Solarbio Life Sciences, R0010) added with cOmplete^TM^, EDTA-free Protease Inhibitor Cocktail (Roche, 4693132001). The lysate was added with 2 x loading buffer (Solarbio Life Sciences, P1019) and measured by the BCA regent (Beijing Applygen Technologies Inc., P1511). The equal protein was resolved on the 10% SDS-PAGE gel and transferred to the Immobilon^®^-P PVDF membrane (Merck Millipore, IPVH00010). The membrane was blocked with 5% Skim Milk (Solarbio Life Sciences, D8340) for 1 hour at room temperature. The primary antibody of STXBP1 (Proteintech, 11459-1-AP) and GAPDH (Proteintech, 60004-1-Ig) were added at 4 ℃ overnight, followed by the corresponding secondary antibody 1 hour at room temperature. The signals were visualized using NcmECL Ultra Stabilized Peroxide Reagent (New Cell & Molecular Biotech, P10300).

### Lactate assay

According to the previous studies 18, 19 , the cells were planted into the 96-well plates. The cells' supernatant was collected and diluted with sterile water. Lactate Assay Kit-WST (Dojindo, L256) was used to detect the lactate level. Based on the concentrations of 10%, 88%, and 2%, the reagents including Dye Mixture Stick Solution, Assay Buffer and Enzyme Solution were mixed. After cultivation for 30 minutes at 37 ℃ condition, the mixture was evaluated at the 450 nm absorbance. P<0.05 was considered as the significant difference.

### ATP analysis

The levels of intracellular ATP were detected using a firefly luciferase-based ATP assay kit (Beyotime Biotechnology, Shanghai, China). In brief, the cells were dissociated using the lysing buffer. Then, the lysates were centrifugated by 12000 × g for 5 minutes at 37 ℃ and were mixed with the ATP detection reagent in the dilution buffer for 5 munities at room temperature. The luminance signaling was measured using the multi-functional microplate reader (Infinite 200 Pro, Tecan, Switzerland). Base on the standard curve, the ATP level was calculated. P<0.05 was determined as the significant difference.

### CCK8 assay

100 μL/well cell suspensions were seeded in the 96-well plates. After cell culture for 24, 48, 72, and 96 hours, cell supernatants were removed, and 90 μL fresh medium and 10 μL reagent from CCK-8 Kit (Dojindo Laboratories) was added. After one hour cultivation, 450 nm absorbance was used to examine cell proliferation.

## Results

### Identification of CCM genes in LUSC

**Figure 1:**
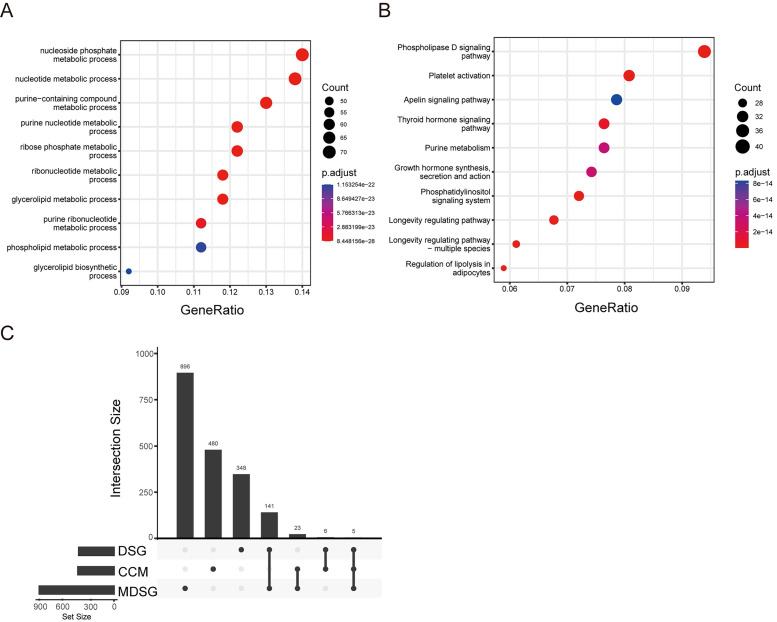
Cancer cell metabolism (CCM) associated pathways. (A) bubble diagram of GO pathway. (B) bubble diagram of KEGG pathway. (C) The gene numbers in the groups including different signature genes (DSG), median DSG (MDSG), CCM, and the numbers of common genes between different groups.

Since the altered cell metabolism is considered a hallmark of cancer, we analyzed status of the CCM related genes in the LUSC. According to the previous study 15, 514 CCM genes were listed in the Stable 1. These genes were enriched in the GO pathways including nucleoside, purine, ribose, and glycerolipid, and KEGG signaling pathways including phospholipase D, platelet activation, apelin, and thyroid hormone, and growth hormone synthesis secretion and action (Figure 1A and B).

### The association of CCM genes with prognosis in LUSC

Next, we screened the genes related to the survival in the LUSC with survival information from TCGA database. 500 of DSG and 1065 of MDSG were identified in LUSC using the previous method 20 and median-dependent method, of which 11 and 28 genes were overlapped with CCM, respectively (Figure 1C, Stable 2, 3, 4 and 5). Both 11 and 28 genes groups contained F10, STXBP1, HAS2, PTEN and MDH1. Among the 11 genes, high expressions of F10, RXRA, PDE1B, ASL, STXBP1, HAS2, PTEN and FHIT were related to shorter survival, and high expressions of MDH1, HPRT1 and LSM2 were related to longer survival (Sfigure 1). Besides, high expressions of ADCY7, CANT1, DPP4, EXT1, FBXW5, SDC4 and SEC31A were associated with shorter survival, and high expressions of UPF3B and CHST7 were associated with longer survival within the 28 genes (Sfigure 2).

### Predictive model of CCM

**Figure 2:**
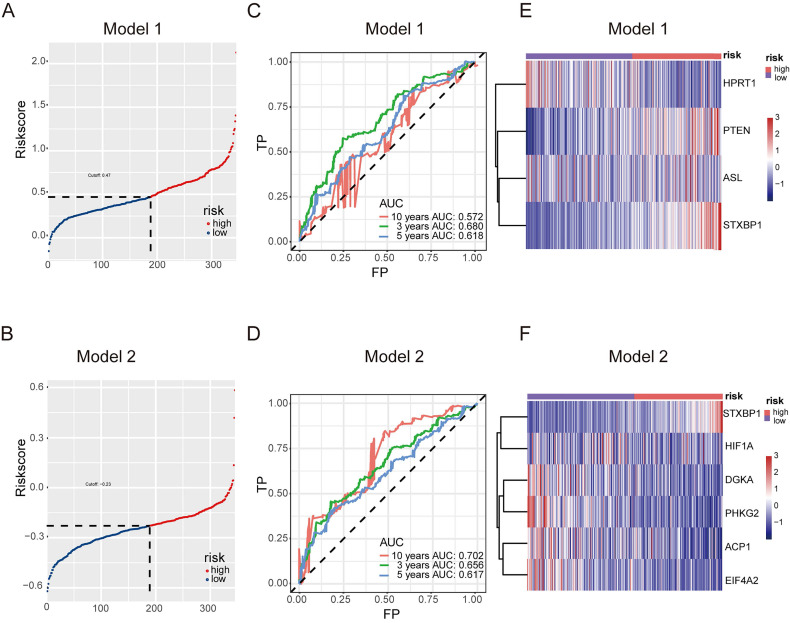
Predictive model for prognosis of LUSC. (A-B) Optimal cutoff determining for patient stratification for model 1 (DSG-CCM, 11 genes as input) (A) and model 2 (MDSG-CCM, 28 genes as input) (B), accordingly. (C-D) ROC curves to evaluate the prognostic value of lasso model derived risk score for model 1 (C) and model 2 (D), accordingly. (E-F) The heatmaps showing the expression of prognostic genes remained in the final models of high risk and low risk groups for model 1 (E) and model 2 (F), accordingly.

The predictive model for prognosis of LUSC was established on the CCM genes by the lasso regression. Those 11 genes and 28 genes were used to create prognostic model 1 and model 2 with the 0.47 and -0.23 as the cutoff value, respectively (Figure 2 A and B). The ROC curve of model 1 was 0.680 for 3 years, 0.618 for 5 years, 0.572 for 10 years, and the ROC curve of model 2 was 0.656 for 3 years, 0.617 for 5 years, 0.702 for 10 years (Figure 2 C and D). The model 1 included ASL, STXBP1, PTEN and HPRT1, and the model 2 contained DGKA, STXBP1, ACP1, PHKG2, EIF4A2, and HIF1A (Figure 2 E and F). The risk scores for model 1 and model 2 were identified as (0.00083
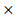
ASL) + (0.015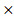 STXBP1) + (0.011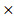 PTEN) + (-0.00074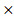 HPRT1) and (-0.001
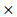
DGKA) + (0.0049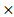 STXBP1) + (-0.00086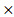 ACP1) + (-0.0079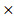 PHKG2) + (-0.0002
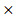
EIF4A2) + (-0.00011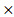 HIF1A) (Stable 6).

**Figure 3:**
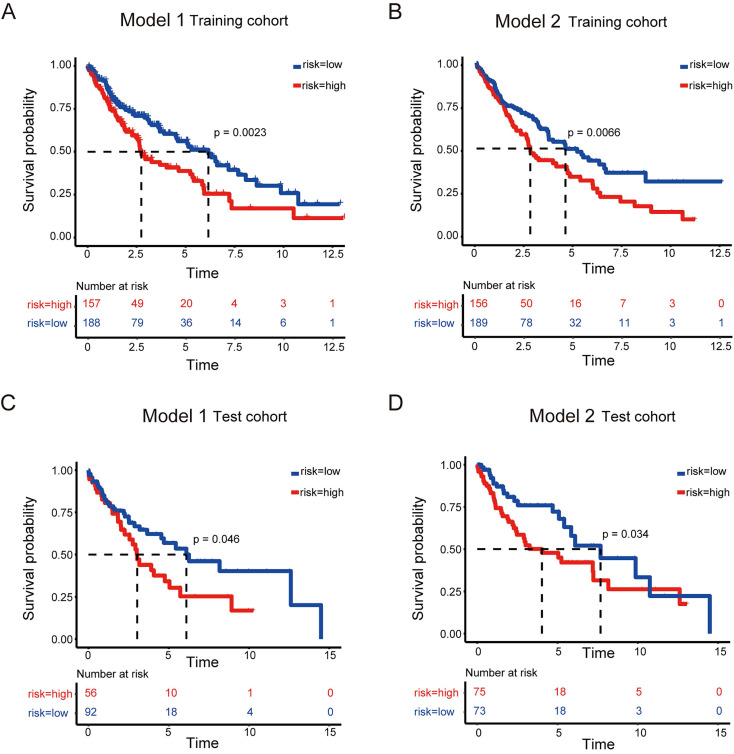
Survival analysis to evaluate model performance in LUSC. (A-B) Survival analysis of the training dataset for model 1 (A) and model 2 (B) of LUSC. (C-D) Survival analysis of the testing dataset for model 1 (C) and model 2 (D) of LUSC.

High-risk LUSC patients showed a poorer survival in the training cohort of both model 1 and model 2 (Figure 3 A and B). Furthermore, high-risk patients had a poorer survival in the test cohort of both model 1 and model 2 (Figure 3 C and D).

Together, these models could predict outcome of LUSC patients and the model 2 showed better ROC effect.

### The correlation of predictive model with immune infiltration

**Figure 4:**
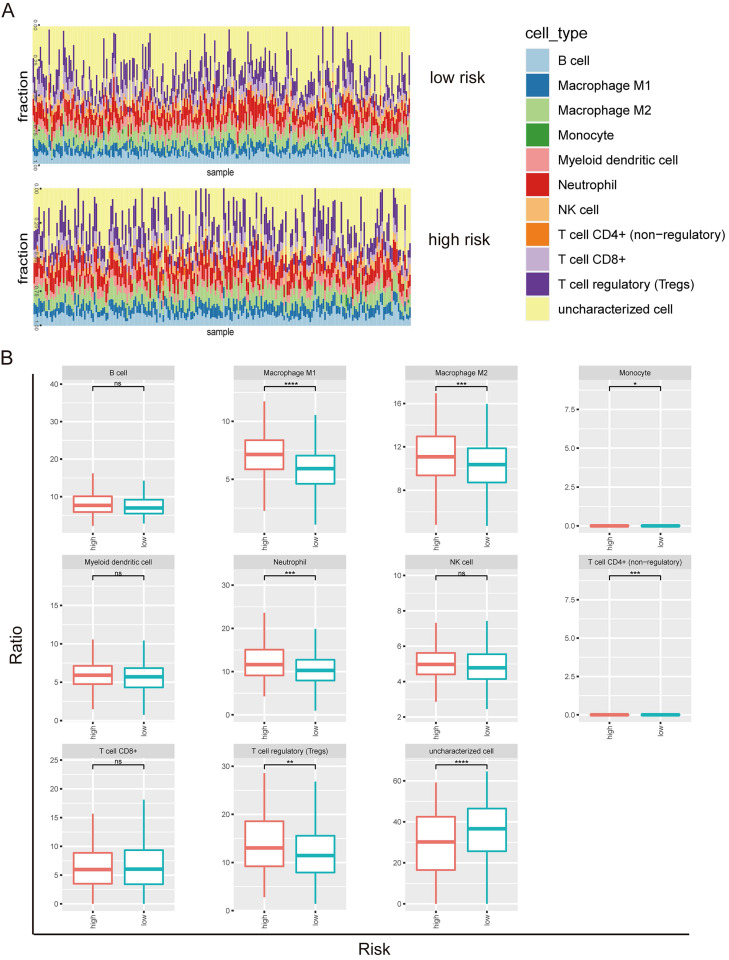
Tumor-infiltrating immune cells in prognostic model 2 in LUSC. (A) Screen of the tumor-infiltrating immune cells in high risk and low risk groups. (B) Distribution of B cell, macrophage M1, macrophage M2, monocyte, myeloid dendritic cell, neutrophil, NK cell, T cell (CD4+), T cell (CD8+), T regulatory cell, and uncharacterized cell in the high and low risks of the model 2.

Next, we investigated the tumor-infiltrating immune cells including B cell, macrophage M1, macrophage M2, monocyte, myeloid dendritic cell, neutrophil, NK cell, T cell (CD4+), T cell (CD8+), T regulatory cell, and uncharacterized cell in the high and low risks of the prognostic model 2 (Figure 4A). B cell, macrophage M1, macrophage M2, myeloid dendritic cell, neutrophil, NK cell, T cell (CD8+), T regulatory cell, and uncharacterized cell types were identified in both high risk and low risk groups (Figure 4B). The enrichments of macrophage M1, macrophage M2, neutrophil, and T regulatory cell types were higher in the high-risk group than the low-risk group. The uncharacterized cell type was less in the high-risk group than the low-risk group.

### STXBP1 was associated with poor outcome of LUSC

**Figure 5:**
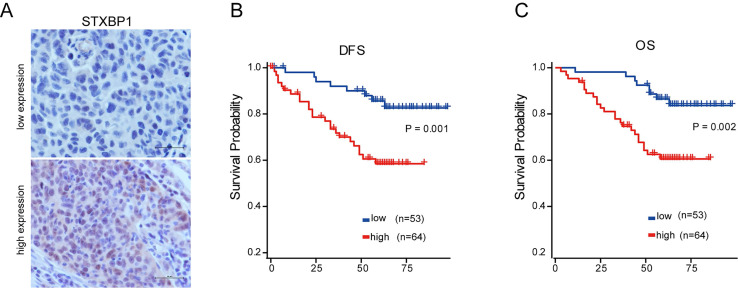
Association of STXBP1 expression with survival of LUSC. (A) Representative images for low (n=53) and high (n=64) STXBP1 expression in tumor tissues from LUSC patients. Scale bar: 50 μM. (B-C) Kaplan-Meier survival curves show the relation between STXBP1 expression levels and disease-free survival (DFS, B) and overall survival (OS, C) in LUSC.

As shown in the figure 2 E and F, only STXBP1 included in the optimized genes of both model 1 and 2. Then, we analyzed the role of STXBP1 in the clinical significance of LUSC by immunohistochemistry. As shown in the Figure 5A, the representative images of low and high STXBP1 expression were demonstrated. Then, these samples were divided into two groups including low and high expression of STXBP1. Furthermore, we analyzed the relationship between this protein expression and clinical characteristics (Table 1). No significant differences were detected between STXBP1 expression and gender, age, or smoking. As we expected, high expression of STXBP1 was related to the advanced stage with significant difference. The Kaplan-Meier curves showed high STXBP1 expression was associated with shorter DFS and OS of LUSC (Figure 5B and C), indicating that STXBP1 could be a predict factor for poor outcome of LUSC.

### STXBP1 related to metabolism and infiltrated immune cells

**Figure 6:**
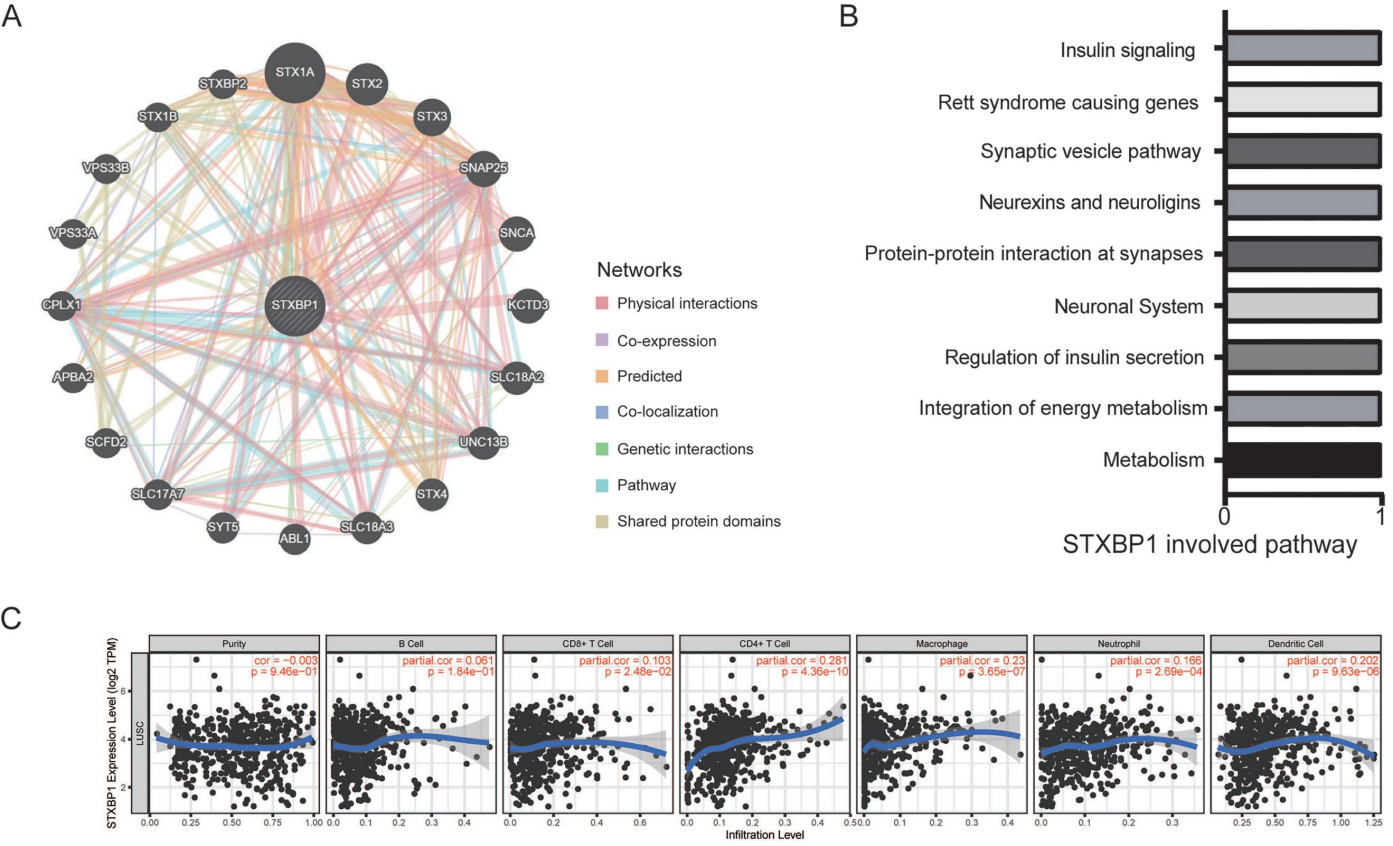
STXBP1 network and infiltrating immune cells in LUSC. (A) Network of STXBP1 and the interacted genes using GeneMANIA. (B) The STXBP1 involved pathways using GeneMANIA. (C) STXBP1 and the tumor-infiltrating immune cells using TIMER.

Then, we found that the genes including STX1A, STX2, STX3, SNAP25, SNCA, and KCTD3 *et al.* interacted with STXBP1 by GeneMANIA analysis (Figure 6A). STXBP1 involved the pathways including metabolism, integration of energy metabolism, regulation of insulin secretion, neuronal system, and protein-protein interaction at synapses by GeneMANIA analysis (Figure 6B). In addition, STXBP1 was related to the infiltrated immune cells including B cell, CD4+ T cell, CD8+ T cell, macrophage, neutrophil, dendritic cell (Figure 6C).

### STXBP1 promoted lactate production, ATP level and cell proliferation of LUSC cells

**Figure 7:**
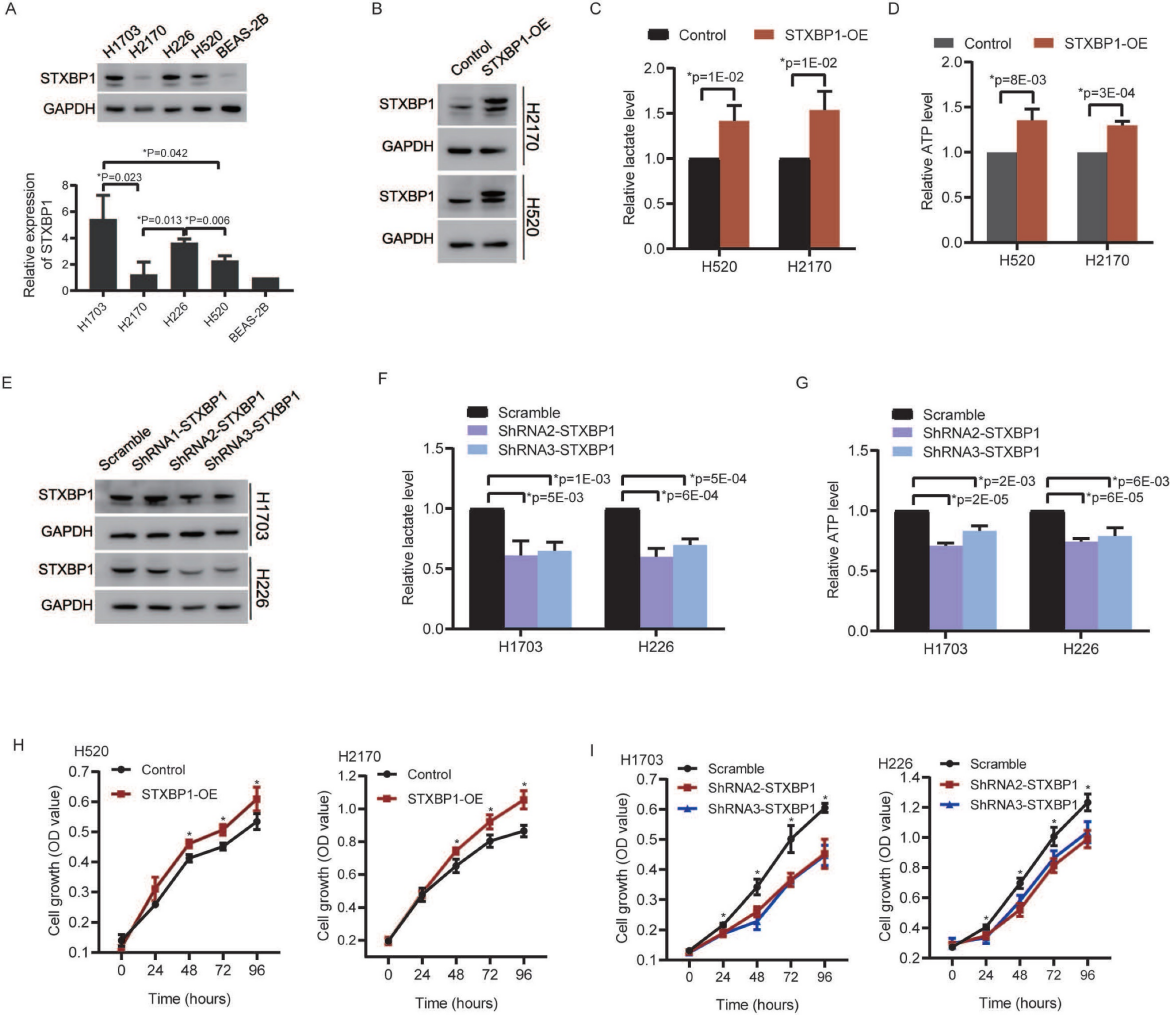
The role of STXBP1 in metabolism and proliferation of LUSC cells. (A) Western blots show STXBP1 in the LUSC cell lines H1703, H2170, H226, and H520, and the human normal lung epithelial cell line BEAS-2B. (B) Western blots show STXBP1 in the H2170 and H520 LUSC cells with overexpression of STXBP1. (C-D) The histograms show the lactate level (C) and ATP level (D) in the H2170 and H520 cells with overexpression of STXBP1. (E) Western blots show STXBP1 in the H1703 and H226 LUSC cells with knock-down of STXBP1 (shRNA1, 2 and 3 targeting this gene). (F-G) The histograms demonstrate the lactate level (F) and ATP level (G) in the H1703 and H226 cells with knock-down of STXBP1. (H-I) The histograms show proliferation of H2170 and H520 cells with overexpression of STXBP1 (H), and H1703 and H226 cells with knock-down of STXBP1 (I).

We analyzed relationships between STXBP1 and the glycolytic metabolism related signals including lactate dehydrogenase (LDH) and facilitative sugar transporter (GLUT) 21, 22. Positive relations were found between STXBP1 and LDHA, GLUT1, and GLUT3 in the LUSC (SFigure 3). There was no significant difference between STXBP1 and LDHB. Furthermore, we investigated the role of STXBP1 in metabolism of lactate and ATP. We screened STXBP1 expression in the LUSC cell lines H1703, H2170, H226, and H520. Compared to the human normal lung epithelial cell line (BEAS-2B), relatively higher expression of this protein was shown in the LUSC cell lines H1703, H2170, H226, and H520 (Figure 7A). Relatively higher expression of STXBP1 were shown in the H1703 and H226 cells, and relatively lower expression of this protein in the H2170 and H520 cells with significant difference (Figure 7A). The H2170 and H520 cells were transfected using lentivirus with overexpression of STXBP1 (Figure 7B). Higher level of lactate and ATP were observed in the H2170 and H520 cells with overexpression of STXBP1 (Figure 7C and D). The shRNA1, 2 and 3 were used to downregulate STXBP1 in the H1703 and H226 cells (Figure 7E). Relatively lower expression of STXBP1 were detected in the cells with shRNA2 and 3 plasmids. Furthermore, lower levels of lactate and ATP were demonstrated in the H1703 and H226 cells with knock-down of STXBP1 (Figure 7F and G). Proliferation was promoted in the H2170 and H520 cells with overexpression of STXBP1 (Figure 7H). Proliferation was inhibited in the H1703 and H226 cells with knock-down of STXBP1 (Figure 7I).

These results suggest STXBP1 involves in the proliferation and metabolism such as lactate and energy production in LUSC.

## Discussion

Cancer cells display specific metabolism that can be considered as therapeutic target 23, 24. It has been reported that not only the tumor itself, but also a metabolic disorder called cancer-associated cachexia drives death for 30% of patients with the advanced cancers 25-27. In current study, the novel findings were as following: ① Based on the lasso modeling, we established the CCM-prognostic model in LUSC; ② Both model 1 and model 2 contained STXBP1 as optimized gene, which was verified to associate with survival of LUSC in our cohort; ③ STXBP1 related to the immune cells including CD4+ T cell, macrophage, and dendritic cell *et al.*, and had the ability to promote lactate and ATP levels, which mainly involved in the cancer metabolism.

For the LUAD, the signature associated with metabolism was constructed to predict prognosis 28. The lasso-based model was used to predict prognosis of LUAD in the immune-associated signatures 29. In the TCGA, we identified 500 genes (DSG) and 1065 genes (MDSG) associated with survival of LUSC using two methods. Among these survival-associated genes, 11 and 28 genes were related to the CCM in DSG and MDSG, respectively. Using Lasso Modeling, these 11 and 28 genes were used to establish CMM-prognostic model 1 and model 2, respectively. For both model 1 and model 2, high-risk patients was related to the shorter survival in the training cohort and the test cohort of LUSC. Furthermore, the ROC curve for 10-year survival of model 2 demonstrated better effect than the model 1, showing an optimal CCM-prognostic model was constructed in LUSC.

The genes including ASL, STXBP1, PTEN and HPRT1 were showed in the model 1, and the genes containing DGKA, STXBP1, ACP1, PHKG2, EIF4A2, and HIF1A were demonstrated in the model 2. STXBP1 was the only one gene in both model 1 and model 2. STXBP1 has been reported to associate with exocytosis like vesicle fusion, priming, docking and membrane fusion 30. In LUAD, high expression of STXBP1 was associated with poor outcome 31. In present study, high expression of this protein was related to poor prognosis (DFS and OS) of LUSC.

Increasing evidences show that the feature and function of immune cells within tumor microenvironment relate with metabolite 10, 13, 32, 33. In the CCM-model 2, more macrophage M1, macrophage M2, neutrophil, and T regulatory cells were enriched in the high-risk group, compared to the low-risk group. Wentao Zhang *et al.* identified the immune-related gene including CD79B, PEBP1, PTK2B, STXBP1, and ZNF671, which were used to establish a proportional hazards regression model in LUAD 34. In glioma, m7G-related genes contained six hub genes including STXBP1, CPLX1, PAB3A, APBA1, RIMS1, and GRIN2B in immune microenvironment 35. These literatures indicated that STXBP1 involved in the immune-related signature. In current study, STXBP1 was found to associate with the infiltrated immune cells including B cell, CD4+ T cell, CD8+ T cell, macrophage, neutrophil, and dendritic cell, suggesting that this gene may play an essential role in regulating immune response.

Under hypoxia condition, the tumor cells likely undergo the essential metabolic progress-glycolysis, that has been known as ''Warburg effect'' with increasing lactate 36. LDH is an important enzyme to regulate the conversion between pyruvate and lactate 21. Since the role of GLUT in glucose metabolism, it is an essential regulator for glycolytic pathway 22. We found positive relation between STXBP1 expression and LDHA, GLUT1, and GLUT3 in the LUSC. Higher levels of lactate and ATP were observed in the STXBP1 overexpressed cells, compared to the control cells. Low levels of lactate and ATP were seen in the LUSC cells with knock-down of STXBP1. Our results indicate that STXBP1 induces ATP energy, that is associated with glycolytic metabolism.

Here, certain limitations were shown. The prognostic model constructions including the training and test cohorts were derived from the TCGA database. Since the scarcity of clinical samples for the LUSC patients, only one key gene-STXBP1 was validated in the clinical samples. Finally, metabolism-associated experiments are limited, more molecular studies are required.

In summary, this study establishes the CCM-associated models to predict prognosis in TCGA LUSC database using lasso multivariable analysis. The CCM-associated model 1 and model 2 could predict survival of LUSC in both training and test cohort. In addition, model 2 showed higher ROC values than model 1, suggesting better effect for predicting. STXBP1, as the only gene in both the model 1 and model 2, was associated with poor survivals of LUSC patients. STXBP1 had positive correlation to the infiltrating immune cells, and promoted the metabolic activities, leading to increased levels of ATP and lactate.

## Supplementary Material

Supplementary figures and tables.

## Figures and Tables

**Table 1:** Clinical variables and STXBP1 expression in LUSC patients (n=117)

Variable	Case no. (%)	STXBP1		*P* value
		Low expression (%)	High expression (%)	
Gender				*0.727*
Male	109 (93.2)	50 (42.7)	59 (50.4)	
Female	8 (6.8)	3 (2.6)	5 (4.3)	
Age (years)				*1*
≤60	53 (45.3)	24 (20.5)	29 (24.8)	
>60	64 (54.7)	29 (24.8)	35 (29.9)	
Smoking history				*0.071*
Yes	109 (93.2)	52 (44.4)	57 (48.7)	
No	8 (6.8)	1 (0.9)	7 (6.0)	
TNM stage				***0.014***
I/II	45 (38.5)	27 (23.1)	18 (15.4)	
III	72 (61.5)	26 (22.2)	46 (39.3)	
