# Supplementary material for: Construction of the metabolism-related models for predicting prognosis and infiltrating immune phenotype in lung squamous cell carcinoma
Source: J Cancer. 2023 Oct 24;14(18):3539–49. doi: 10.7150/jca.86942 (PMC10647197; doi:10.7150/jca.86942)
Supplement: Supplementary file 1 — Supplementary figures and tables. [file jcav14p3539s1.pdf]

Sfigure legend

SFigure 1 Kaplan-Meier survival curves of 11 genes in LUSC. The survival curves were analyzed in LUSC cases using GEPIA.

SFigure 2 Kaplan-Meier survival curves of 23 genes in LUSC. The survival curves were analyzed in LUSC cases using GEPIA.

SFigure 3 Relations between STXBP1 and LDHA, LDHD, GLUT1, and GLUT3 in LUSC using GEPIA.

A

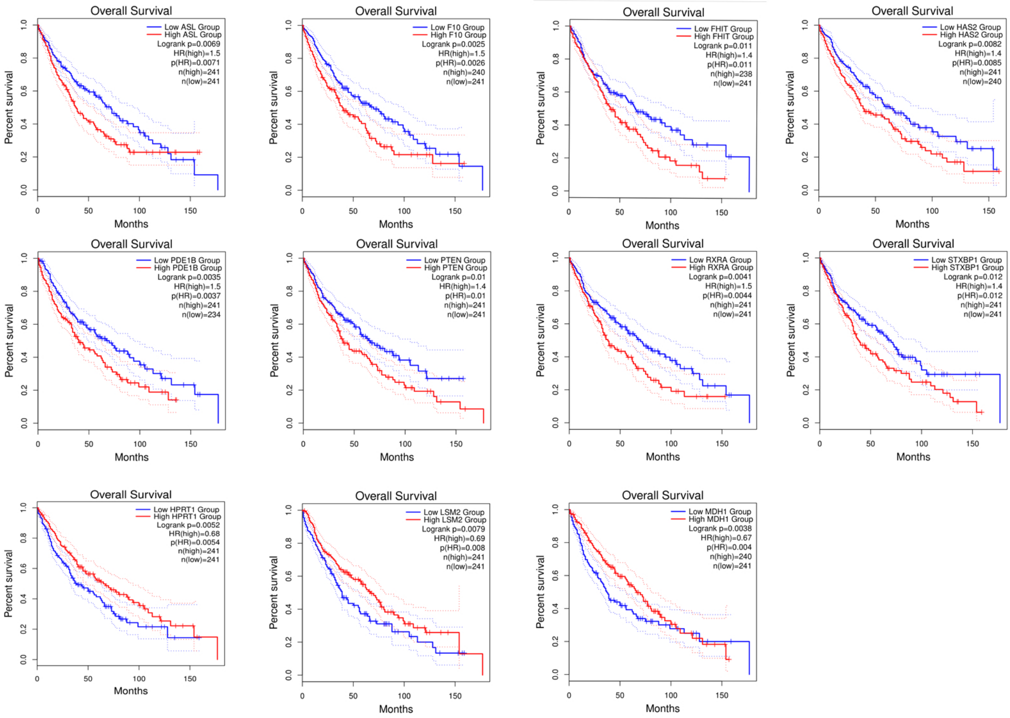
Overall Survival
Low ASL Group
High ASL Group
Logrank p=0.0069
HR(high)=1.5
p(HR)=0.0071
n(high)=241
n(low)=241
Overall Survival
Low F10 Group
High F10 Group
Logrank p=0.0025
HR(high)=1.5
p(HR)=0.0026
n(high)=240
n(low)=241
Overall Survival
Low FHIT Group
High FHIT Group
Logrank p=0.011
HR(high)=1.4
p(HR)=0.011
n(high)=238
n(low)=241
Overall Survival
Low HAS2 Group
High HAS2 Group
Logrank p=0.0082
HR(high)=1.4
p(HR)=0.0085
n(high)=241
n(low)=240
Overall Survival
Low PDE1B Group
High PDE1B Group
Logrank p=0.0035
HR(high)=1.5
p(HR)=0.0037
n(high)=241
n(low)=234
Overall Survival
Low PTEN Group
High PTEN Group
Logrank p=0.01
HR(high)=1.4
p(HR)=0.01
n(high)=241
n(low)=241
Overall Survival
Low RXRA Group
High RXRA Group
Logrank p=0.0041
HR(high)=1.5
p(HR)=0.0044
n(high)=241
n(low)=241
Overall Survival
Low STXBP1 Group
High STXBP1 Group
Logrank p=0.012
HR(high)=1.4
p(HR)=0.012
n(high)=241
n(low)=241
Overall Survival
Low HPRT1 Group
High HPRT1 Group
Logrank p=0.0052
HR(high)=0.68
p(HR)=0.0054
n(high)=241
n(low)=241
Overall Survival
Low LSM2 Group
High LSM2 Group
Logrank p=0.0079
HR(high)=0.69
p(HR)=0.008
n(high)=241
n(low)=241
Overall Survival
Low MDH1 Group
High MDH1 Group
Logrank p=0.0038
HR(high)=0.67
p(HR)=0.004
n(high)=240
n(low)=241
Percent survival
Months

A

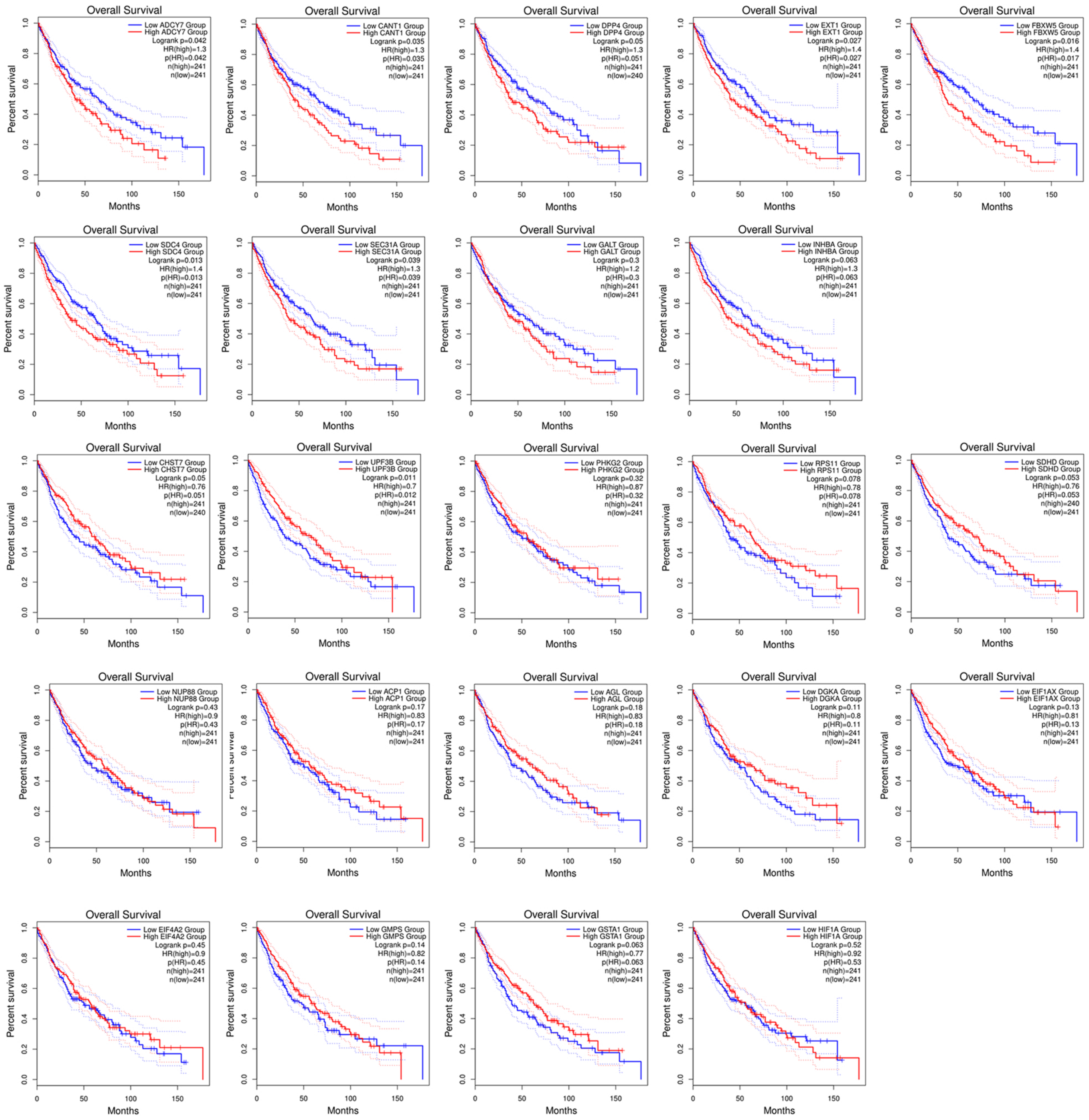
Overall Survival
Low ADCY7 Group
High ADCY7 Group
Logrank p=0.042
HR(high)=1.3
p(HR)=0.042
n(high)=241
n(low)=241
Overall Survival
Low CANT1 Group
High CANT1 Group
Logrank p=0.035
HR(high)=1.3
p(HR)=0.035
n(high)=241
n(low)=241
Overall Survival
Low DPP4 Group
High DPP4 Group
Logrank p=0.05
HR(high)=1.3
p(HR)=0.051
n(high)=241
n(low)=240
Overall Survival
Low EXT1 Group
High EXT1 Group
Logrank p=0.027
HR(high)=1.4
p(HR)=0.027
n(high)=241
n(low)=241
Overall Survival
Low FBXW5 Group
High FBXW5 Group
Logrank p=0.016
HR(high)=1.4
p(HR)=0.017
n(high)=241
n(low)=241
Overall Survival
Low SDC4 Group
High SDC4 Group
Logrank p=0.013
HR(high)=1.4
p(HR)=0.013
n(high)=241
n(low)=241
Overall Survival
Low SEC31A Group
High SEC31A Group
Logrank p=0.039
HR(high)=1.3
p(HR)=0.039
n(high)=241
n(low)=241
Overall Survival
Low GALT Group
High GALT Group
Logrank p=0.3
HR(high)=1.2
p(HR)=0.3
n(high)=241
n(low)=241
Overall Survival
Low INHBA Group
High INHBA Group
Logrank p=0.063
HR(high)=1.3
p(HR)=0.063
n(high)=241
n(low)=241
Overall Survival
Low CHST7 Group
High CHST7 Group
Logrank p=0.05
HR(high)=0.76
p(HR)=0.051
n(high)=241
n(low)=240
Overall Survival
Low UPF3B Group
High UPF3B Group
Logrank p=0.011
HR(high)=0.7
p(HR)=0.012
n(high)=241
n(low)=241
Overall Survival
Low PHKG2 Group
High PHKG2 Group
Logrank p=0.32
HR(high)=0.87
p(HR)=0.32
n(high)=241
n(low)=241
Overall Survival
Low RPS11 Group
High RPS11 Group
Logrank p=0.078
HR(high)=0.78
p(HR)=0.078
n(high)=241
n(low)=241
Overall Survival
Low SDHD Group
High SDHD Group
Logrank p=0.053
HR(high)=0.76
p(HR)=0.053
n(high)=240
n(low)=241
Overall Survival
Low NUP88 Group
High NUP88 Group
Logrank p=0.43
HR(high)=0.9
p(HR)=0.43
n(high)=241
n(low)=241
Overall Survival
Low ACP1 Group
High ACP1 Group
Logrank p=0.17
HR(high)=0.83
p(HR)=0.17
n(high)=241
n(low)=241
Overall Survival
Low AGL Group
High AGL Group
Logrank p=0.18
HR(high)=0.83
p(HR)=0.18
n(high)=241
n(low)=241
Overall Survival
Low DGKA Group
High DGKA Group
Logrank p=0.11
HR(high)=0.8
p(HR)=0.11
n(high)=241
n(low)=241
Overall Survival
Low EIF1AX Group
High EIF1AX Group
Logrank p=0.13
HR(high)=0.81
p(HR)=0.13
n(high)=241
n(low)=241
Overall Survival
Low EIF4A2 Group
High EIF4A2 Group
Logrank p=0.45
HR(high)=0.9
p(HR)=0.45
n(high)=241
n(low)=241
Overall Survival
Low GMPS Group
High GMPS Group
Logrank p=0.14
HR(high)=0.82
p(HR)=0.14
n(high)=241
n(low)=241
Overall Survival
Low GSTA1 Group
High GSTA1 Group
Logrank p=0.063
HR(high)=0.77
p(HR)=0.063
n(high)=241
n(low)=241
Overall Survival
Low HIF1A Group
High HIF1A Group
Logrank p=0.52
HR(high)=0.92
p(HR)=0.53
n(high)=241
n(low)=241
Percent survival
Months

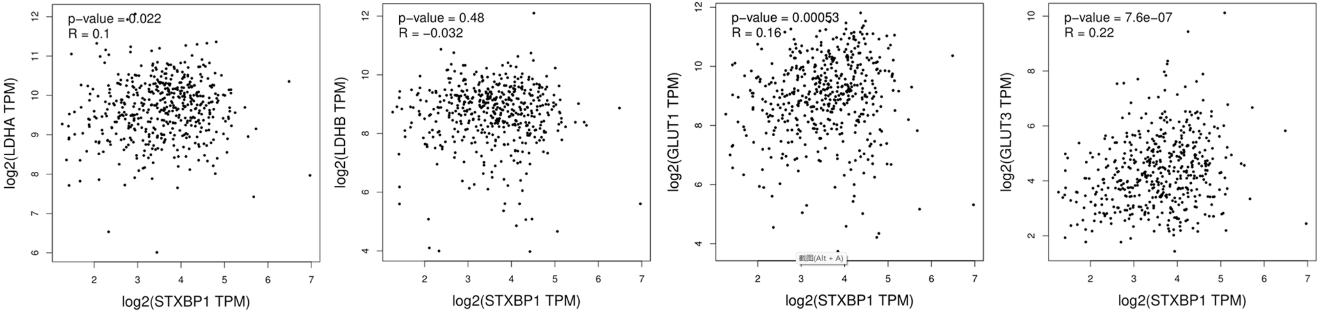
p−value = 0.022
R = 0.1
log2(LDHA TPM)
log2(STXBP1 TPM)
p−value = 0.48
R = −0.032
log2(LDHB TPM)
log2(STXBP1 TPM)
p−value = 0.00053
R = 0.16
log2(GLUT1 TPM)
log2(STXBP1 TPM)
截图(Alt + A)
p−value = 7.6e−07
R = 0.22
log2(GLUT3 TPM)
log2(STXBP1 TPM)

Stable 1 cancer cell metabolism (CCM) in LUSC

Gene Symbol	Gene ID	Gene Symbol	Gene ID	Gene Symbol	Gene ID	Gene Symbol	Gene ID	Gene Symbol	Gene ID	Gene Symbol	Gene ID
MED24	9862	CHST7	56548	PRKAG2	51422	FBXW5	54461	PIK3C2B	5287	PIK3C3	5289
INPP4A	3631	PRPS1	5631	GCK	2645	NPR2	4882	ADCY1	107	GSTP1	2950
MED17	9440	EIF3H	8667	TYRP1	7306	PSMC2	5701	INPPL1	3636	AKR1B1	231
PABPC1	26986	POLR3A	11128	PSMD3	5709	ADCY9	115	SIN3A	25942	MTAP	4507
LMAN1	3998	ALLC	55821	ASPA	443	PRKAA2	5563	SHMT1	6470	PRODH	5625
CNOT4	4850	GUCY1A2	2977	ENO3	2027	AGL	178	POLE	5426	PRMT5	10419
PTGS1	5742	CCT8	10694	APOA5	116519	ALB	213	ZFP36L1	677	PPP2CB	5516
PNPLA3	80339	ACAN	176	RPS13	6207	PSMD6	9861	NCOR2	9612	TUSC3	7991
PSMA6	5687	EIF5A2	56648	DAO	1610	POLR2H	5437	MTOR	2475	CAV1	857
NUP93	9688	NOS3	4846	MVK	4598	PRKACG	5568	GNAI2	2771	INPP4B	8821
PSMD11	5717	ABCA1	19	EIF2B1	1967	FXN	2395	RXRA	6256	MAPK14	1432
NUP107	57122	KCNC2	3747	GAPDH	2597	HPRT1	3251	GNA15	2769	CCNC	892
XRN1	54464	DPAGT1	1798	PRKAB1	5564	PDIA3	2923	ADCY2	108	NIT2	56954
EEF1B2	1933	GALNTL6	442117	COL4A3BP	10087	SMG8	55181	NCOA3	8202	PROC	5624
RPS15	6209	CHST1	8534	ARSB	411	LALBA	3906	PLD2	5338	ARG1	383
GOT1	2805	ALOX12B	242	PPP2CA	5515	TUBA1A	7846	ADCY3	109	CTGF	1490
CAT	847	ALOX15B	247	PRKAR2A	5576	TK1	7083	ADCY8	114	INHA	3623
RPL5	6125	SEPHS2	22928	FBXO6	26270	POLR2G	5436	NAT2	10	GNMT	27232
ACO1	48	MUC16	94025	RPE65	6121	BMP1	649	PRKD1	5587	ZFP36	7538
UPF3B	65109	ST6GALNAC3	256435	AMPD1	270	NPR1	4881	NCOA6	23054	DNMT1	1786
EIF2S2	8894	PDE4B	5142	GALE	2582	FABP4	2167	SMG5	23381	EIF2S1	1965
GNAI1	2770	DPYD	1806	APOA1	335	PLA2G1B	5319	PRKAB2	5565	GRP	2922
TPTE2	93492	ACSL5	51703	FBXL5	26234	HAS2	3037	NCOR1	9611	PLCE1	51196
ARNTL	406	PI4KA	5297	ABCG2	9429	C1GALT1C1	29071	ALDH2	217	GLIPR1	11010
LDHA	3939	MGAM	8972	EIF2B2	8892	POLR1C	9533	SDC4	6385	ANP32A	8125
CLOCK	9575	ABCB11	8647	NFYB	4801	PAH	5053	MED12	9968	ARF1	375
UGT2A3	79799	PNLIPRP2	5408	ADCY7	113	SMN1	6606	WWTR1	25937	PTGES	9536
KIF13A	63971	BGN	633	ACADS	35	ALG14	199857	OMD	4958	FAU	2197
SEC31A	22872	TNPO1	3842	EXOSC9	5393	AGXT	189	CANT1	124583	ALOX15	246
LUM	4060	DIS3	22894	RPS10	6204	DCP2	167227	GNA11	2767	PRKCD	5580
RPS2	6187	ARFGEF2	10564	NUP153	9972	ADCY5	111	PPP2R1A	5518	NUDT2	318
SLC44A3	126969	MUC17	140453	SLC10A2	6555	PSMD1	5707	DNMT3A	1788	PSMC3	5702
PIP5K1A	8394	GPC5	2262	POLR1B	84172	ADCY6	112	ACSL3	2181	RRM1	6240
ITPKB	3707	GPC6	10082	F10	2159	RPL4	6124	PIK3R1	5295	SDC2	6383
RQCD1	9125	CD38	952	NR1D1	9572	POLD4	57804	IDH2	3418	BSG	682
CCT5	22948	PDK4	5166	TPH1	7166	SPHK1	8877	XPO1	7514	PLA2G16	11145
SEC24D	9871	PRKAR2B	5577	ADCY4	196883	POLR2L	5441	NCOA1	8648	MTHFR	4524
ACAD8	27034	PDK2	5164	LDLR	3949	DPM3	54344	KDSR	2531	UPP1	7378
HPD	3242	NOS2	4843	PRKCSH	5589	CALR	811	NCOA2	10499	GSTT1	2952
MAT2A	4144	IYD	389434	APOE	348	AKAP5	9495	SDHC	6391	PPARA	5465
GUSB	2990	ACPP	55	PRKAA1	5562	TH	7054	GNAQ	2776	PLCD1	5333
PAIP1	10605	CD44	960	EIF5A	1984	F2	2147	GMPS	8833	DPP4	1803
EIF1AX	1964	CP	1356	GUCY2D	3000	NQO1	1728	RPN1	6184	CNOT7	29883
TBL1XR1	79718	RRM2B	50484	CSNK1G2	1455	POLR2A	5430	PAFAH1B2	5049	GPX3	2878
RPS27	6232	F7	2155	RDH5	5959	GLUD2	2747	GPHN	10243	RPS29	6235
PLCB1	23236	POLD1	5424	SMPD2	6610	PROS1	5627	GNAS	2778	NAT6	24142
SRPR	6734	SPHK2	56848	AGT	183	MAPK11	5600	EP300	2033	HYAL1	3373
MUC6	4588	CTSA	5476	DGKB	1607	PSMD13	5719	RPL22	6146	PPP2R1B	5519
WBSCR17	64409	DGKA	1606	STXBP1	6812	POLR1D	51082	ATIC	471	VBP1	7411
GNG2	54331	ELAVL1	1994	DPM2	8818	PPP1CC	5501	EIF4A2	1974	RPL10	6134
GPX5	2880	FECH	2235	POLR1E	64425	EIF4EBP1	1978	ACSL6	23305	ABCB1	5243
AMY2B	280	PDK3	5165	ITPKA	3706	PRKAR1B	5575	PLAUR	5329	MED23	9439
POM121	9883	POLR1A	25885	HADHB	3032	UROS	7390	HEXB	3074	ACACA	31
CNOT3	4849	MAOB	4129	SLC5A6	8884	MAOA	4128	SREBF1	6720	EEF1A1	1915
VCAN	1462	GUCY2C	2984	HNRNPD	3184	PIK3R4	30849	PIGU	128869	HDAC3	8841
MED29	55588	PRKACA	5566	MTTP	4547	SULT1A1	6817	EIF3E	3646	INHBA	3624
NUP133	55746	PLD1	5337	FURIN	5045	PSMD12	5718	SLC5A5	6528	XDH	7498
PLA2G10	8399	DGKD	8527	PARN	5073	GK	2710	HSPB1	3315	PPM1L	151742
SLC44A1	23446	POLD3	10714	RPS11	6205	HSPA1B	3304	NUP88	4927	YWHAZ	7534
LRP2	4036	RAPGEF3	10411	SLC2A5	6518	LSM2	57819	PFDN5	5204	YWHAB	7529
AACS	65985	EIF3I	8668	RPL11	6135	NUP62	23636	ITPA	3704	FHIT	2272
SI	6476	GCKR	2646	PRKACB	5567	VDAC1	7416	RAP1B	5908	FH	2271
ITPR3	3710	B4GALT1	2683	PFDN2	5202	GALT	2592	MUC4	4585	SDHB	6390
PLCB4	5332	SLC9A1	6548	CERS2	29956	PPP2R2A	5520	FASN	2194	GPC3	2719
OGN	4969	RAPGEF4	11069	ACP1	52	AMY1A	276	TYMS	7298	EXT2	2132
UGT2B10	7365	PHGDH	26227	EIF2B5	8893	MIF	4282	SNRPE	6635	EXT1	2131
LTA4H	4048	COMT	1312	DGKQ	1609	UGT1A9	54600	P4HB	5034	SDHD	6392
GNB4	59345	POLR2F	5435	GLUD1	2746	GSTA1	2938	ENTPD5	957	CREBBP	1387
DGUOK	1716	HMOX1	3162	DGKZ	8525	BCKDHA	593	RPL7A	6130	FBXW7	55294
TPO	7173	CERK	64781	RPS3	6188	INS	3630	ACTB	60	IDH1	3417
ACADM	34	POLE2	5427	HNMT	3176	PDE3B	5140	PIK3CG	5294	TP53	7157
MUC5B	727897	MTHFD1	4522	EIF4E	1977	MLXIPL	51085	LSM1	27257	PTGS2	5743
PDE1B	5153	PSMC1	5700	FBXO4	26272	ALOX5	240	PLCG2	5336	DNMT3B	1789
MOCS1	4337	PSMB5	5693	MGAT5	4249	MAPKAPK2	9261	TPR	7175	HIF1A	3091
MED1	5469	TRIB3	57761	PDK1	5163	GSPT2	23708	NUP214	8021	CA9	768
ASL	435	AHCY	191	RANBP2	5903	GLA	2717	PPARG	5468	RAP1A	5906
NUP210	23225	POLA1	5422	DGKE	8526	G6PD	2539	CDX2	1045	NME1	4830
DMGDH	29958	GUCY2F	2986	PRKCA	5578	NT5C3A	51251	DDX6	1656	YAP1	10413
SLCO1B1	10599	USP11	8237	CDK19	23097	POLR2B	5431	NFYA	4800	AKR1B10	57016
CD36	948	DGKH	160851	SLC16A1	6566	PIK3CB	5291	PAX6	5080	PRKAR1A	5573
GALNT5	11227	PSMD7	5713	RPL30	6156	DGKG	1608	MDH1	4190	STK11	6794
PIK3C2G	5288	CA2	760	PHKG2	5261	CAD	790	PPAP2A	8611	PIK3CA	5290
INHBE	83729	GPI	2821	SMG1	23049	HSPA8	3312	RORA	6095	AKT1	207
GUK1	2987	POLR2I	5438	DGKI	9162	EIF4G1	1981	CA12	771	CTNNB1	1499
ABCG8	64241	PIK3R2	5296	PFKFB1	5207	PIK3R3	8503	ENO1	2023	PTEN	5728
GALNT13	114805	OGDH	4967	APOA2	336	PLCG1	5335				

Stable 2 different signature genes (DSG) in LUSC

Gene Symbol	P-Value (Survival	Gene Symbo	P-Value	Gene Symbol	P-Value	Gene Symbol	P-Value	Gene Symbol	P-Value
VRK1	2.60E-05	SERPIND1	1.71E-03	CCDC51	3.87E-03	FGA	6.06E-03	RPL7P9	8.67E-03
PARVA	2.89E-05	C11orf45	1.74E-03	RXRA	3.87E-03	ZNF749	6.18E-03	HEG1	8.74E-03
PCDHGA11	3.49E-05	LNP1	1.75E-03	CADM3	3.90E-03	RP11-265N6.2	6.18E-03	SPRYD4	8.78E-03
TRIM58	3.53E-05	APBB2	1.76E-03	MYO1G	3.94E-03	HOXB4	6.24E-03	TNFRSF12A	8.80E-03
RP11-196I18.3	4.12E-05	ANKRD18C	1.77E-03	AC133528.2	3.95E-03	PCDHA5	6.27E-03	NOL3	8.83E-03
PAPPA	4.93E-05	FES	1.77E-03	THBS1	3.97E-03	ASL	6.35E-03	MME	8.84E-03
HIGD1B	1.50E-04	SYNJ2BP-C	1.78E-03	KIF16B	3.97E-03	SEMA7A	6.37E-03	RGN	8.86E-03
RP11-47A8.5	1.68E-04	MGRN1	1.79E-03	AOC3	3.98E-03	LIPK	6.40E-03	9-Sep	8.88E-03
SNAI1	1.91E-04	MACROD2	1.80E-03	HOXD-AS2	4.00E-03	GAPDHP72	6.49E-03	RP11-535M15	8.93E-03
SMAD5-AS1_1	2.08E-04	RP11-60H5	1.89E-03	MAGI2-AS3	4.01E-03	FN1	6.54E-03	HNRNPA1P12	8.94E-03
RP11-47G11.2	2.11E-04	CHMP4C	1.92E-03	PCDHA2	4.03E-03	ESR1	6.64E-03	PPM1N	9.01E-03
PCDHGA12	2.15E-04	VAV2	1.94E-03	PON2	4.04E-03	FLRT3	6.64E-03	TMEM255B	9.01E-03
AP5S1	2.19E-04	DCDC2	1.95E-03	GPIHBP1	4.10E-03	RPL6P27	6.66E-03	RP11-1100L3.	9.02E-03
ISM2	2.47E-04	AC084809.1	2.00E-03	CITED2	4.13E-03	FMO6P	6.70E-03	SEZ6L2	9.09E-03
CD151	2.55E-04	PYGB	2.10E-03	SERPINE1	4.18E-03	AC012442.5	6.72E-03	CALML6	9.10E-03
OR2W3	2.59E-04	MEF2B	2.11E-03	RP11-466H18.1	4.21E-03	KATNAL1	6.76E-03	AC007952.6	9.12E-03
CLEC4M	2.62E-04	RP5-998N2	2.13E-03	AC055733.1	4.30E-03	RP11-86H7.7	6.78E-03	AC005162.5	9.15E-03
ELANE	2.73E-04	MEGF11	2.14E-03	LOXL2	4.37E-03	TNFRSF10B	6.80E-03	ADAT1	9.16E-03
RP11-248M19.1	2.84E-04	B3GNT7	2.15E-03	MYADM	4.39E-03	C5orf17	6.83E-03	RP11-685B14.	9.19E-03
FAM65A	3.02E-04	AC007326.1	2.22E-03	C7orf55	4.42E-03	LINC01089	6.83E-03	RP5-1180E21.	9.25E-03
RP11-535M15.1	3.16E-04	PDLIM3	2.23E-03	PDLIM1P4	4.42E-03	RP11-680F8.1	6.85E-03	MAP1LC3B	9.26E-03
RP11-392P7.6	3.46E-04	LGI2	2.25E-03	RP11-204M4.2	4.43E-03	TAS2R2P	6.94E-03	RP11-69L16.4	9.26E-03
ACVRL1	3.48E-04	RP11-547D	2.29E-03	ZNF727	4.43E-03	TGM2	6.94E-03	ROS1	9.27E-03
MBNL2	3.55E-04	F10	2.34E-03	LDAH	4.46E-03	CTD-2245F17.	6.95E-03	TMA7	9.28E-03
HORMAD2-AS1	3.79E-04	PITX3	2.34E-03	VDR	4.46E-03	WDR78	6.99E-03	RP11-161H23	9.31E-03
HELLS	3.81E-04	RP11-1046B	2.41E-03	ZNF423	4.50E-03	GPR182	6.99E-03	RPS4XP2	9.34E-03
NEK6	4.08E-04	RP11-36C2	2.43E-03	PDE1A	4.60E-03	BHMT2	7.02E-03	GPR160	9.39E-03
RP11-102M11.1	4.64E-04	RP11-125H	2.43E-03	FSTL3	4.60E-03	RP11-1E11.1	7.02E-03	LPL	9.41E-03
CD14	5.16E-04	RP11-386G	2.43E-03	FAM46B	4.62E-03	AC016739.2	7.06E-03	RP5-1061H20	9.41E-03
ST6GALNAC4	5.16E-04	CHMP2B	2.46E-03	APOL6	4.63E-03	AIFM1	7.07E-03	WDPCP	9.42E-03
PCDH1	5.36E-04	SLC38A8	2.49E-03	FAM103A1	4.64E-03	SLC9A7P1	7.07E-03	TRIB1	9.44E-03
ZDHHC7	5.44E-04	JPH1	2.50E-03	PTPRU	4.64E-03	TFCP2L1	7.08E-03	LINC00968	9.47E-03
SGCA	5.50E-04	SPATA6	2.51E-03	ZFP36L2	4.65E-03	CRISPLD2	7.10E-03	BMP2	9.50E-03
TCF21	5.53E-04	CTC-459F4	2.52E-03	GGTLC1	4.70E-03	TMEM92	7.15E-03	PINK1	9.50E-03
SAMD11	5.60E-04	FAM46A	2.52E-03	AC079922.2	4.71E-03	SMIM22	7.17E-03	CPB2	9.52E-03
SLC9A9	5.79E-04	HLA-H	2.53E-03	FGF7	4.73E-03	STOX1	7.20E-03	SRRT	9.54E-03
TMEM8A	5.88E-04	C9orf66	2.53E-03	MORC2	4.74E-03	MOCOS	7.21E-03	RPL7P28	9.62E-03
PTX3	5.94E-04	TRIM22	2.59E-03	HOGA1	4.75E-03	FBLIM1	7.21E-03	ACTG2	9.64E-03
FOXD4L4	6.54E-04	FCN3	2.59E-03	MMP8	4.78E-03	UGT2B4	7.26E-03	RP11-672L10.	9.68E-03
MYL3	6.70E-04	VPS28	2.62E-03	SERP1	4.78E-03	PCBD2	7.27E-03	KRT17P5	9.68E-03
KRT42P	6.82E-04	PTBP3	2.62E-03	EFCAB2	4.83E-03	GGCT	7.27E-03	TRIM45	9.70E-03
C11orf96	7.13E-04	SAMD14	2.62E-03	PM20D1	4.89E-03	RP11-57C13.6	7.28E-03	HCG4P5	9.77E-03
ERH	7.72E-04	IRS4	2.63E-03	PEX19	4.90E-03	CTC-510F12.4	7.28E-03	EDNRB	9.85E-03
RNF103-CHMP3	7.84E-04	NPNT	2.64E-03	PLA2G5	4.94E-03	MCCC1-AS1	7.29E-03	NFKB2	9.87E-03
RP11-74C1.2	7.92E-04	RPS4XP22	2.68E-03	MMP23A	4.95E-03	RPS6KA1	7.35E-03	SLC26A9	9.88E-03
SOD3	8.10E-04	KCNIP2-AS	2.68E-03	AC074286.1	4.97E-03	MMP28	7.38E-03	GABRA5	9.90E-03
FOXD1-AS1	8.27E-04	LRPAP1	2.70E-03	FEZF1	4.97E-03	APIP	7.40E-03	APP	9.92E-03
TRIM55	8.34E-04	UNC5A	2.74E-03	HPRT1	5.03E-03	MICALCL	7.40E-03	AC004453.8	9.92E-03
ADAMTS17	8.46E-04	RP11-428K	2.75E-03	RP11-434H6.6	5.06E-03	SLC4A1APP1	7.45E-03	ANXA5	9.94E-03
SBK3	8.47E-04	ONECUT3	2.78E-03	ZNF567	5.06E-03	LSM2	7.49E-03	RP11-114H24.	9.96E-03
ZNF835	8.49E-04	SNRPEP4	2.79E-03	IST1	5.08E-03	TRABD2B	7.51E-03	PTGIS	9.97E-03
ANK2	8.76E-04	PTGIR	2.79E-03	CTSD	5.17E-03	GCOM1	7.53E-03	FKBP3	9.99E-03
KB-226F1.2	8.83E-04	AC104532.4	2.81E-03	LRCH1	5.22E-03	RP11-356B19.	7.55E-03	RP11-347I19.8	1.00E-02
FNDC3A	8.97E-04	TIMM8AP1	2.83E-03	RP11-173M11.2	5.24E-03	CTSL	7.58E-03	RP11-13E5.2	1.01E-02
PRKCDBP	9.26E-04	OPCML	2.84E-03	ADGRD1	5.24E-03	DPP6	7.60E-03	PTPN21	1.01E-02
HSPB7	9.31E-04	CNTNAP4	2.84E-03	JPH2	5.29E-03	KNDC1	7.61E-03	NPDC1	1.01E-02
CTD-2541J13.2	9.56E-04	TLDC1	2.86E-03	RP11-20F18.1	5.29E-03	PCDHA10	7.63E-03	CTB-113P19.4	1.02E-02
CRACR2B	9.60E-04	FLT4	2.88E-03	ZP1	5.31E-03	PCDHA12	7.65E-03	LINC01481	1.02E-02
XXbac-BPG116M	9.71E-04	RP11-67C2	2.93E-03	PPAPDC3	5.32E-03	FGG	7.68E-03	HNRNPA1P48	1.02E-02
RP11-546O6.4	9.74E-04	HDAC11	3.00E-03	RP5-943J3.2	5.33E-03	RIN2	7.69E-03	PTEN	1.02E-02
RP11-468E2.1	9.78E-04	NDUFA6	3.03E-03	SNX29	5.35E-03	TMEM204	7.72E-03	RP3-523K23.2	1.02E-02
AGPAT3	1.02E-03	FGF8	3.03E-03	SLC16A4	5.36E-03	RP11-95G6.1	7.79E-03	SLC51B	1.02E-02
ALDH7A1	1.05E-03	CD163L1	3.05E-03	NKAPL	5.39E-03	TFPI2	7.79E-03	HILS1	1.03E-02
MSR1	1.06E-03	MAST4-AS	3.05E-03	PKIG	5.40E-03	FBLN2	7.82E-03	AP006285.2	1.04E-02
KCTD1	1.10E-03	TIMM8B	3.07E-03	CTD-3157E16.1	5.43E-03	TMX4	7.84E-03	PTGDS	1.04E-02
RP11-146F11.1	1.11E-03	TRIM5	3.18E-03	PDGFRB	5.45E-03	CD5L	7.85E-03	AC090044.2	1.05E-02
LONRF3	1.13E-03	ITGA3	3.20E-03	ATP13A5	5.47E-03	RP11-166D19.	7.86E-03	RP11-54D18.4	1.05E-02
ACTA2	1.15E-03	PGA3	3.20E-03	GSTA9P	5.47E-03	DBP	7.86E-03	CRYBB3	1.05E-02
PCDH12	1.16E-03	ARMCX7P	3.21E-03	TNFRSF8	5.48E-03	STAC2	7.91E-03	IFT57	1.05E-02
AD000864.6	1.20E-03	RPL3P4	3.23E-03	RP11-460N11.2	5.49E-03	RP11-475C16.	7.92E-03	ASCL4	1.05E-02
TNFSF12	1.23E-03	HTR2B	3.25E-03	HGF	5.50E-03	SCG5	7.93E-03	ARC	1.06E-02
RP11-298I3.5	1.25E-03	NPM1P21	3.26E-03	DACT3	5.56E-03	CPA2	7.98E-03	RN7SL731P	1.06E-02
RP11-973D8.5	1.28E-03	ZNF876P	3.31E-03	OVOL1-AS1	5.56E-03	TMC4	8.00E-03	SLC35F2	1.06E-02
TEX21P	1.29E-03	RP11-173C	3.34E-03	MICAL2	5.57E-03	RALGAPA2	8.01E-03	TRAPPC2	1.06E-02
RP11-317L10.1	1.29E-03	MEIS3P1	3.34E-03	ATP6V1F	5.59E-03	ANO3	8.01E-03	CTD-2626G11	1.07E-02
HRASLS	1.33E-03	ZNF589	3.36E-03	SUMF1	5.59E-03	CEACAM6	8.10E-03	RP11-25I15.1	1.07E-02
GADD45B	1.34E-03	PDE1B	3.36E-03	SUGT1P2	5.63E-03	CASP4	8.13E-03	C17orf50	1.07E-02
MT1L	1.35E-03	CEP295NL	3.37E-03	CFB	5.64E-03	HAS2	8.17E-03	KNTC1	1.07E-02
RP11-543P15.1	1.36E-03	PCDHGA10	3.39E-03	RP11-700J17.2	5.67E-03	KBTBD6	8.21E-03	FHIT	1.08E-02
PKNOX2	1.38E-03	SHPK	3.40E-03	GALNT2	5.67E-03	RP5-940J5.9	8.22E-03	RP11-464F9.2	1.08E-02
RP11-153M7.3	1.39E-03	RP11-778D	3.44E-03	MED12L	5.67E-03	CCDC177	8.23E-03	FBN1	1.08E-02
AC234582.1	1.41E-03	RP11-76C10	3.46E-03	LA16c-380H5.5	5.68E-03	AC144450.2	8.23E-03	KRTAP4-1	1.08E-02
PXN	1.43E-03	ZER1	3.49E-03	RNF181	5.69E-03	PCDH8	8.23E-03	GNPTG	1.08E-02
PSMF1	1.45E-03	BMP5	3.50E-03	RHD	5.71E-03	GLYATL2	8.25E-03	VPS35	1.08E-02
SNHG21	1.50E-03	STAP1	3.51E-03	RP11-48B3.3	5.76E-03	EDEM1	8.36E-03	RP11-168F9.2	1.08E-02
NT5E	1.53E-03	MYL2	3.52E-03	RWDD2B	5.77E-03	L1CAM	8.39E-03	RP11-198M15	1.08E-02
ATP5L	1.53E-03	ZNF415	3.53E-03	RP11-506H20.1	5.79E-03	SMCP	8.47E-03	RP11-302I18.1	1.09E-02
ELMO3	1.56E-03	ABCC9	3.53E-03	TDGF1	5.85E-03	LMO4	8.47E-03	TMEM217	1.10E-02
EMCN	1.56E-03	CCDC68	3.54E-03	IL3RA	5.86E-03	RP11-216B9.6	8.51E-03	TINAGL1	1.10E-02
RP11-613M5.2	1.57E-03	PHF5A	3.54E-03	RP3-412A9.16	5.87E-03	PWWP2A	8.52E-03	ANGPTL2	1.10E-02
GOLGA8J	1.59E-03	DUSP18	3.61E-03	REL	5.88E-03	NAV2	8.54E-03	PDE3A	1.11E-02
SEC23B	1.61E-03	PCDHGB5	3.64E-03	RP11-903H12.5	5.89E-03	IGHV3-71	8.55E-03	TBC1D1	1.11E-02
CLEC11A	1.64E-03	RNF8	3.65E-03	P2RX4	5.92E-03	GRAPL	8.57E-03	AC093850.2	1.11E-02
USP39	1.65E-03	CD47	3.66E-03	SNRPC	5.98E-03	FAM72B	8.61E-03	NACC2	1.11E-02
CCDC73	1.66E-03	MDH1	3.72E-03	LATS2	5.99E-03	RP1-159A19.4	8.62E-03	XXbac-BPG24	1.12E-02
ITIH3	1.67E-03	FAM196B	3.73E-03	RP11-6N17.3	6.00E-03	POC5	8.63E-03	DES	1.12E-02
TMEM231	1.67E-03	AAED1	3.77E-03	CD300LG	6.00E-03	RP11-54A9.1	8.63E-03	C4BPB	1.12E-02
CLDN5	1.68E-03	ASF1B	3.78E-03	HID1	6.01E-03	SFTA3	8.63E-03	FHL5	1.12E-02
GMPSP1	1.69E-03	CYBA	3.79E-03	PTGR2	6.03E-03	MIR31HG	8.63E-03	STXBP1	1.12E-02
PAX9	1.71E-03	RP11-95M1	3.85E-03	ST3GAL5-AS1	6.03E-03	CD68	8.64E-03	RP11-848P1.3	1.13E-02

Stable 3 median DSG (MDSG)) in LUSC

GeneID	HR	GeneID	HR	GeneID	HR	GeneID	HR	GeneID	HR	GeneID	HR	GeneID	HR	GeneID	HR	GeneID	HR
SAMD11	1.4	AL078621.3	0.75	CTBP1	1.5	FHL5	1.3	BAG4	0.75	FOLR2	1.5	PSPC1	0.76	MT1E	1.4	PTGIS	1.4
TNFRSF18	0.75	DBI	0.7	ZFYVE28	1.4	FAXC	0.75	PLPP5	0.7	PDE2A	1.6	ATP12A	0.72	MT1A	1.4	SNAI1	1.6
TMEM240	1.3	GPR39	1.4	RNF4	1.4	WASF1	0.69	NSD3	0.75	LRRC32	1.3	WASF3	1.4	AC012181.1	1.4	AL354993.2	0.72
MIB2	1.3	ZRANB3	0.74	ADD1	1.3	DSE	1.4	AC087623.2	0.72	PAK1	0.71	FLT1	1.3	CMTM3	1.4	RBM38	1.3
PEX10	1.3	LYPD6B	0.68	WFS1	1.4	ROS1	1.4	VDAC3	0.73	ALG8	0.75	POSTN	1.3	TRADD	1.4	HMGB1P1	1.3
SMIM1	1.6	RPRM	0.73	TADA2B	1.6	SERINC1	1.4	RPL37P6	0.71	FAM181B	0.74	KBTBD7	0.67	E2F4	1.4	ZBP1	1.4
AL031847.1	1.4	ITGB6	1.4	HSP90AB2P	0.73	STX7	1.4	UBXN2B	1.4	FZD4	1.4	TSC22D1	1.3	ELMO3	1.5	PMEPA1	1.3
TNFRSF8	1.4	LINC01806	0.68	TAPT1	1.4	SNORA33	0.72	SDCBP	1.4	CASP4	1.5	AL138693.1	0.75	RIPOR1	1.4	APCDD1L	1.4
FBLIM1	1.3	DPP4	1.4	QDPR	1.4	TCF21	1.5	AC100814.2	0.68	SLC35F2	1.3	LRCH1	1.6	ACD	1.4	PPP1R3D	0.72
HSPB7	1.4	AC019181.1	0.72	LGI2	1.6	SGK1	0.74	TRAM1	1.3	TIMM8B	0.64	PSME2P2	0.74	EDC4	1.4	RBBP8NL	0.7
CPLANE2	1.3	GAD1	0.74	STIM2.AS1	1.3	AL356234.2	0.75	JPH1	0.6	SDHD	0.69	FNDC3A	1.5	PSKH1	1.5	TPGS1	1.4
ARHGEF10L	1.3	AC078883.3	0.67	APBB2	1.4	AL590617.2	0.74	GDAP1	0.68	CCDC84.DT	0.73	PHF11	1.3	SLC12A4	1.4	FSTL3	1.5
PLA2G5	1.6	AC016739.1	0.69	FIP1L1	0.76	ADGRG6	1.4	AC115837.1	0.7	HMBS	0.74	THSD1	1.3	PDF	1.3	FAM174C	0.65
PINK1	1.4	NFE2L2	0.72	HOPX	1.7	TAB2	1.4	PKIA	1.3	THY1	1.3	HNRNPA3P5	0.75	TERF2	1.3	GAMT	0.74
ALPL	1.3	DNAJC19P5	0.75	STAP1	1.4	LINC01615	1.4	CHMP4C	1.4	JHY	1.4	H3P36	0.73	WWP2	1.4	MBD3	0.75
AL021154.1	0.67	AC079305.2	0.75	TMPRSS11A	0.73	TBP	0.72	HNRNPA1P4	0.64	PKNOX2	1.5	PTMAP5	0.73	VAC14	1.5	ATP8B3	0.71
MACO1	1.3	AC019080.1	0.66	AC025244.1	0.74	MRM2	0.75	RIPK2	1.4	GSEC	1.3	DNAJC3	1.5	ZFP1	1.4	SPPL2B	0.74
RPS6KA1	1.4	FKBP7	1.4	CDKL2	1.4	EIF3B	0.7	OSR2	0.73	C11orf45	1.4	MBNL2	1.6	TMEM231	1.5	GADD45B	1.4
FCN3	1.4	COQ10B	1.4	ANTXR2	1.5	CYTH3	1.4	SNX31	0.73	JAM3	1.4	RAP2A	1.4	ADAT1	1.5	ZNF57	0.71
BX293535.1	0.75	C2orf69	0.74	SEC31A	1.4	COL28A1	1.4	ZFPM2	1.6	THYN1	0.74	STK24.AS1	0.73	ATMIN	1.4	DOHH	0.71
TINAGL1	1.5	AC010731.3	0.74	ARHGAP24	0.75	AC006042.1	1.3	EXT1	1.3	LINP1	1.4	H2AZP3	0.74	C16orf46	1.6	GIPC3	1.3
ADGRB2	1.3	KANSL1L	1.4	C4orf36	1.3	CRPPA	0.71	ENPP2	1.4	SFTA1P	1.4	TPP2	1.4	MBTPS1	1.4	TBXA2R	1.4
SYNC	1.3	AC079834.2	0.69	SNCA	0.7	NUP42	0.7	HAS2	1.3	PPIAP31	0.75	ING1	1.3	CRISPLD2	1.5	VMAC	0.75
GJA4	1.3	AC010980.2	0.65	AP002026.1	0.74	GGCT	0.76	FAM83A	1.3	AL365203.2	1.4	ATP11A	1.3	RNU1.103P	1.4	ARHGEF18	1.5
MYCL	0.75	CT75	0.68	ADH7	0.72	SLC25A5P5	0.7	TRIB1	1.4	ITGB1	1.4	F10	1.5	FOXL1	1.4	MCEMP1	1.3
AC098484.2	0.75	KCNE4	1.5	NPNT	0.68	LSM5	0.74	DENND3	1.4	FZD8	0.75	ABHD4	0.76	MAP1LC3B	1.4	ADAMTS10	1.4
AL357079.1	0.74	ITM2C	1.3	PLA2G12A	1.4	AC018645.3	0.72	GPIHBP1	1.4	BMS1	0.75	AL132780.2	0.65	PIEZO1	1.4	ZNF699	1.3
PLK3	1.4	HTR2B	1.4	INTU	1.4	KBTBD2	1.3	GLI4	0.74	ZNF32.AS1	0.74	PABPN1	0.75	TUBB3	1.3	RPS4XP22	0.66
BTBD19	1.4	B3GNT7	1.5	PPP1R14BP3	0.71	POU6F2.AS2	0.73	SCRIB	1.4	SLC16A9	0.69	DHRS4L2	0.71	RPH3AL	1.4	PDE4A	1.4
CYP4B1	1.3	MSL3P1	0.72	H3P16	0.69	INHBA	1.4	PUF60	1.5	JMJD1C	1.4	TM9SF1	1.3	MYO1C	1.5	KRI1	0.73
ACOT11	1.4	HES6	0.65	USP38	1.3	SNORA5C	0.75	HSF1	1.3	AL513534.3	0.74	RIPK3	1.4	AC002316.1	1.4	TIMM29	0.69
TTC4	1.4	EDEM1	1.6	SFRP2	1.3	ABCA13	0.64	DGAT1	1.4	HKDC1	1.3	AL390334.1	0.69	SHPK	1.3	KANK2	1.4
ITGB3BP	0.75	CRELD1	1.4	FGG	1.5	FIGNL1	0.75	ZNF250	1.3	AC010997.4	0.71	AL139023.1	0.72	CTNS	1.4	ECSIT	0.75
RPS7P4	0.74	FBLN2	1.4	GLRB	1.3	MRPS17	0.75	DMRT2	0.74	SFTPA2	1.3	ARHGAP5	0.74	AC132942.1	0.7	CNN1	1.4
IFI44L	1.3	NKIRAS1	0.72	AC080188.2	0.7	SNORA22	0.7	IFNWP19	1.3	SFTPA1	1.4	SFTA3	1.4	MINK1	1.4	TRIR	0.74
CLCA2	0.66	CMTM7	1.3	VEGFC	1.4	GTF2IP23	0.75	CYP4F26P	0.74	ANXA11	1.5	NKX2.1	1.4	SLC52A1	0.64	PRDX2	0.75
LMO4	0.68	WDR48	1.4	STOX2	0.74	DNAJC30	0.75	GALT	1.3	CCSER2	1.5	PAX9	0.64	NUP88	0.71	FARSA	0.72
KYAT3	0.74	AC099560.2	0.64	PDLIM3	1.5	MAGI2.AS3	1.4	AL512604.1	0.68	RPS3AP5	0.74	PNN	0.74	ALOX12.AS1	0.68	NFIX	0.74
GBP6	0.73	SNRK	1.3	AC093909.6	0.75	CFAP69	1.3	ZBTB5	0.75	PTEN	1.4	HNRNPUP1	0.71	BCL6B	1.3	C19orf53	0.71
GBP1P1	0.71	ANO10	1.5	BASP1	1.3	GTPBP10	0.7	MIR4477B	0.73	FRA10AC1	0.72	RTRAF	0.68	ZBTB4	1.4	AC005785.2	0.72
GEMIN8P4	0.74	CCR5AS	1.3	AC010343.1	0.76	AC002075.2	0.73	AL161787.1	0.72	SLC35G1	0.72	PELI2	0.69	TNFSF12	1.4	HAUS8	0.74
AGL	0.75	PTH1R	1.4	AC114956.1	0.72	DLX6	0.71	CDK20	1.4	HELLS	0.65	TMEM260	0.75	AC005224.3	0.72	RPL39P38	0.75
AC118553.1	0.65	AC104447.1	1.4	ITGA1	1.3	DLX5	0.67	AUH	1.4	CYP2C18	0.75	TIMM9	0.75	MEIS3P1	1.6	CRTC1	1.3
SLC35A3	0.73	PTPN23	1.4	RPL26P19	0.73	SDHAF3	0.75	CENPP	0.72	ARHGAP19	0.75	AL049873.1	0.75	AC015922.2	1.3	COPE	1.3
SASS6	0.72	ZNF589	0.69	MAST4.AS1	0.74	PDAP1	0.73	ANKRD19P	0.75	FRAT2	0.72	SIX1	0.68	UBB	1.6	AC139769.1	1.4
TRMT13	0.71	CCDC51	0.72	MRPS27	1.3	ZNF655	0.74	AL589843.1	1.6	ANKRD2	0.74	TMEM30B	0.74	DRG2	1.3	AC006504.7	0.69
KIAA1324	0.72	GPX1	1.3	AC008972.2	1.3	ZSCAN25	0.7	ALG2	1.3	HOGA1	0.63	AL359220.1	0.72	AC107983.1	0.73	PLEKHF1	1.3
KCND3	1.4	GMPPB	1.4	POC5	1.4	TFR2	0.72	INVS	1.3	CRTAC1	1.4	HIF1A	0.74	UBE2SP2	0.75	C19orf12	1.5
WNT2B	0.66	IP6K1	1.4	JMY	0.73	SLC12A9	0.7	PTBP3	1.4	PYROXD2	1.3	HSPE1P2	0.73	UBBP4	1.4	SNORA68B	1.3
PPM1J	0.74	MST1R	1.4	MTX3	0.75	SERPINE1	1.4	KIF12	1.3	NFKB2	1.3	YBX1P1	0.72	SDF2	0.73	GRAMD1A	1.4
TSPAN2	1.4	SEMA3B	1.4	LUCAT1	1.4	PRKRIP1	0.74	TNFSF15	1.5	TRIM8	1.3	RDH11	0.75	SUZ12P1	0.72	COX7A1	1.3
TRIM45	0.73	CACNA2D2	1.3	ERAP1	1.5	ALKBH4	0.73	PAPPA	1.5	AL391121.1	1.6	EXD2	0.75	CCL2	1.4	AC008649.1	0.76
NBPF9	1.3	C3orf18	1.4	AC133134.1	0.73	LRRC17	1.3	PHF19	1.4	ARL3	1.4	ERH	0.7	SLFN13	1.5	AC022144.1	0.74
H2BC20P	0.69	AC006252.1	0.7	TSLP	0.75	AC007683.1	0.75	TTLL11	1.3	DUSP5	1.4	AC005476.2	0.69	AP2B1	1.3	CYP2S1	0.75
AL391069.2	0.75	AC096887.2	0.75	TMED7	1.5	DNAJC2	0.69	NEK6	1.4	BBIP1	1.4	PTGR2	0.66	MMP28	1.4	CEACAM6	1.3
TUFT1	0.75	DENND6A	1.4	PTMAP2	0.74	KMT2E.AS1	0.73	STXBP1	1.5	TCF7L2	1.4	SAMD15	1.4	EPOP	0.76	RPS19	0.7
AL365436.1	0.75	PSMC1P1	0.66	AC119150.1	0.72	AC004884.2	0.73	AK1	1.4	VWA2	1.4	STON2	0.74	RARA.AS1	1.3	XRCC1	0.75
C2CD4D.AS1	1.4	HNRNPA3P6	0.75	AC106786.1	0.7	CDHR3	0.75	AL356481.1	1.4	CACUL1	1.4	GALC	1.3	TMEM99	0.72	ERCC2	0.75
BX470102.1	0.75	CHMP2B	1.3	ALDH7A1	1.6	RPL7P32	0.69	AL356481.3	1.3	PLEKHA1	0.75	AL049834.1	1.3	KRT42P	0.67	ERCC1	0.68
MUC1	1.4	ST3GAL6	1.3	CSF2	1.3	FEZF1	0.65	ZDHHC12	1.4	EEF1AKMT2	0.71	AL122020.1	0.73	VAT1	1.4	PNMA8B	1.3
KHDC4	0.73	LNP1	0.7	KIF3A	1.4	FEZF1.AS1	0.73	IER5L	1.4	PTPRE	1.3	RPS6KA5	0.7	SP6	1.4	PTGIR	1.5
CADM3	1.3	AC073861.1	0.7	SHROOM1	1.4	LRRC4	0.75	NTMT1	1.4	SPRN	0.73	NDUFB1	0.71	COPZ2	0.74	DACT3	1.4
LINC01133	0.68	CD47	1.7	PCBD2	0.73	FLNC	1.4	USP20	1.4	WASH8P	1.4	TMEM251	0.74	HOXB2	1.3	MAMSTR	0.69
CCDC190	0.76	IFT57	1.4	MTCYBP18	0.73	AC009275.1	1.3	NCS1	1.5	WNT5B	0.75	SYNE3	1.4	HOXB5	1.4	RPS11	0.71
LINC00626	0.75	ABHD10	0.75	PROB1	0.72	KLRG2	0.69	ABL1	1.3	FBXL14	1.5	BDKRB2	1.3	AC103702.2	1.4	BCL2L12	0.72
F5	1.3	IGSF11	0.76	DNAJC18	1.3	TCAF1P1	1.4	LAMC3	1.4	AC125807.1	0.69	VRK1	0.74	ITGA3	1.5	AC006942.1	1.3
KIFAP3	1.5	AC112484.3	0.71	CD14	1.5	AOC1	1.7	PLPP7	1.4	PARP11	0.66	RPL3P4	0.72	SGCA	1.6	CLEC11A	1.4
FMO6P	0.73	RAB43	1.4	PCDHGB7	1.3	FASTK	0.72	POMT1	1.3	CCND2	0.72	AL162151.2	0.7	TMEM92	1.5	ZNF175	1.4
PFN1P1	0.75	PLXND1	1.5	PCDH12	1.5	ACE2	0.74	TSC1	1.4	CD9	0.72	SNORA28	0.75	MAP3K3	1.4	ZNF808	1.7
SUCO	1.3	AC117409.1	0.68	SPINK5	0.7	PDHA1	0.76	RALGDS	1.3	CD27.AS1	0.68	ATP5MPL	0.7	ERN1	1.4	AC022150.2	1.5
RPS29P5	0.67	NPHP3	0.75	ABLIM3	1.3	EIF1AX	0.74	SURF4	1.5	GNB3	0.76	MIR4538	1.3	ABCA5	0.74	ZNF160	1.4
AL136454.1	0.71	TMEM108	1.3	RPL7P1	0.67	KLHL15	0.67	SARDH	1.4	CD163L1	1.4	RPL41P2	0.71	SUMO2	0.75	ZNF415	1.4
SNORA77	1.4	AC108727.1	0.72	TNIP1	1.4	FTLP2	0.68	VAV2	1.5	NECAP1	1.3	GREM1	1.3	UNC13D	1.4	ZNF765	1.3
NUAK2	1.4	RNF7	0.74	CCDC69	1.4	DYNLT3	0.69	BRD3OS	1.4	GPRC5A	1.3	AC018868.1	0.75	SRSF2	0.71	MYADM	1.6
SLC26A9	1.4	SLC9A9	0.68	MFAP3	1.3	MID1IP1.AS1	0.75	NOTCH1	1.4	GPRC5D.AS1	0.73	THBS1	1.5	AFMID	0.67	TMC4	1.6
HSD11B1	1.3	DIPK2A	0.67	FABP6	1.3	RPS2P55	0.7	AGPAT2	1.5	GPRC5D	0.69	AC037198.1	1.4	CANT1	1.6	CDC42EP5	1.4
AC092017.1	0.7	PFN2	0.73	SLU7	1.4	GAPDHP65	0.71	PHPT1	1.7	HTR7P1	0.75	AC025166.1	0.72	CARD14	0.74	IL11	1.4
SLC30A1	1.3	SERP1	0.75	AC026403.1	0.72	CHST7	0.75	FBXW5	1.3	AC010168.2	0.71	GABPB1	0.76	ARHGDIA	1.4	ZNF71	1.3
TP53BP2	1.4	EIF2A	0.72	GABRP	1.4	RGN	1.3	PTGDS	1.4	RPL7P6	0.71	GABPB1.AS1	0.73	THOC1	0.66	ESS2	0.7
MRPL55	0.74	SIAH2.AS1	0.65	FBXW11	1.3	NUS1P1	1.4	CLIC3	1.4	PLEKHA5	0.75	TMOD2	1.4	GAPLINC	1.4	AC000068.2	0.74
FAM96AP2	0.75	LINC02006	0.75	PDLIM7	1.4	HSPB1P2	0.72	DPP7	1.5	ABCC9	1.3	RPS3AP6	0.75	RNMT	0.69	AIFM3	0.75
GALNT2	1.4	MME	1.5	AC106795.2	0.74	CLCN5	0.74	NELFB	1.4	SOX5	0.76	C15orf61	0.71	AC090772.5	0.75	MAPK1	0.69
ARV1	0.73	SLC33A1	0.73	AC136632.1	0.73	AL139396.1	0.75	PKP3	1.3	TMTC1	0.73	ARIH1	0.75	DSC3	0.63	RAB36	1.3
RPS7P3	0.7	GMPS	0.75	N4BP3	1.5	MAGED2	1.4	PNPLA2	1.4	AC024940.3	0.67	RCN2	0.73	SLC39A6	0.74	SNRPD3	0.75
COA6.AS1	0.74	PTX3	1.6	MAPK9	1.3	EDA2R	1.4	CRACR2B	1.5	AC084824.6	0.74	ADAMTS7	1.4	MOCOS	1.4	GGT1	1.3
TARBP1	0.75	GFM1	0.71	FLT4	1.4	FAM155B	0.71	CD151	1.4	KIF21A	0.74	ABHD17C	0.75	RPL7AP66	0.75	CRYBB3	0.73
AL603825.1	0.64	RPL23AP42	0.69	DUSP22	1.7	RPS23P8	0.69	AC068580.4	1.4	LRRK2.DT	1.3	SNHG21	0.7	MTCO2P2	0.65	TTC28	0.7
ACP1	0.74	AC078802.1	0.75	F13A1	1.5	NONO	0.66	CTSD	1.5	LRRK2	1.4	WHAMM	0.74	CTIF	1.5	CHEK2	0.72
ADI1	0.69	GPR160	0.76	DSP	0.74	HDAC8	0.76	TNNI2	1.3	PLEKHA8P1	1.4	C15orf40	0.66	SMAD7	1.3	Z93930.3	0.69
TAF1B	0.72	PHC3	0.75	AL136162.1	0.74	PBDC1	0.75	TRIM22	1.4	ANO6	1.5	MRPL46	0.73	MBD2	0.74	NF2	0.74
GRHL1	0.72	AC026355.2	0.71	GPLD1	0.74	ZNF711	0.75	CAVIN3	1.5	VDR	1.4	POLG	0.75	SNRPGP2	0.73	MORC2	0.66
RAB10	0.69	ACTL6A	0.72	H2BC9	0.73	EIF4A1P10	0.69	SMPD1	1.4	ASIC1	0.7	ADAMTS17	0.61	SERPINB13	0.72	AC005005.3	0.68
EIF2B4	0.71	SOX2	0.76	ZNF192P1	0.72	PCDH19	0.71	ILK	1.4	LINC00592	0.74	SPATA41	0.68	FAUP1	0.69	PIK3IP1.AS1	0.74
WDR43	0.74	MCCC1	0.72	HLA.H	1.4	TRMT2B	0.76	ZNF214	1.3	KRT74	0.67	SNRNP25	0.71	NRSN2.AS1	1.5	PATZ1	0.7
COX7A2L	0.73	YEATS2	0.74	SFTA2	1.4	ARMCX5	0.72	STK33	1.4	KRT78	0.69	PGAP6	1.5	TBC1D20	1.4	AL096701.1	0.74
ZFP36L2	1.4	ABCC5	0.73	AL671883.2	1.5	AL117327.1	1.4	RPL23AP65	0.74	ITGA5	1.3	TPSB2	1.4	AP5S1	1.3	PRR14L	0.75
ACYP2	1.3	CLCN2	0.69	VARS1	1.3	CHRDL1	1.3	MICAL2	1.4	MMP19	1.4	SNORD60	0.64	MIR103A2	1.4	Z74021.1	0.73
AC007098.1	0.74	RPL4P4	0.7	FKBPL	0.62	LRCH2	1.3	PARVA	1.5	DGKA	0.74	TRAF7	1.4	CDS2	1.6	MIR6819	0.74
WDPCP	0.65	TRA2B	0.69	AL451165.2	0.61	UPF3B	0.7	SPON1	1.4	SPRYD4	0.68	AC006111.2	0.75	MACROD2	1.5	FOXRED2	0.76
MDH1	0.67	EIF4A2	0.72	FANCE	0.74	THOC2	0.75	COPB1	1.3	C12orf56	0.69	MGRN1	1.5	FLRT3	1.3	NOL12	0.6
UGP2	0.76	BCL6	0.65	SRSF3	0.69	STAG2	0.74	C11orf96	1.7	GNS	1.4	NUDT16L1	0.74	DSTN	1.4	TNRC6B	0.71
AC012368.3	0.75	TPRG1	0.75	TMEM217	1.4	ZNF280C	0.74	CD82	1.5	C12orf29	0.76	NPIPA5	0.71	SEC23B	1.3	SLC25A17	0.71
C2orf42	0.72	FGF12	0.71	RNF8	0.73	AL009174.1	0.73	DDB2	0.72	BTG1	0.73	RPL7P47	0.73	RPL12P12	0.68	CHADL	0.73
AC016700.2	0.69	PLAAT1	0.7	FO393411.1	0.75	SMIM10L2A	0.73	MTCH2	0.73	AC012085.1	0.74	ITPRIPL2	1.5	SLC24A3	1.4	CSDC2	1.3
FAM136A	0.73	AC080129.2	0.68	CLIC5	1.3	VMA21	0.64	GLYATL2	0.66	NTN4	1.5	AC002550.2	0.73	KIZ	1.5	SREBF2.AS1	0.7
TGFA	1.4	TMEM44.AS1	0.75	ENPP4	1.3	SLC6A8	0.75	FAM111A.DT	1.3	ACTR6	0.74	IL4R	1.4	AL035258.1	0.68	NDUFA6	0.72
ACTG2	1.4	XXYLT1	0.71	CYP39A1	1.4	L1CAM	1.4	STX3	1.3	C12orf75	0.76	TUFM	0.72	GZF1	1.4	AC254562.3	0.72
INO80B	0.68	ACAP2.IT1	0.73	AL109918.1	0.72	BRCC3	0.73	SYT7	1.4	SSH1	1.3	CDIPTOSP	0.72	CST3	1.7	NUP50	0.73
LBX2.AS1	1.4	PPP1R2	0.74	GSTA1	0.67	TNFRSF10B	1.5	EHD1	1.4	GLTP	0.72	KCTD13	0.75	PYGB	1.5	AL121672.2	0.75
AC093162.1	0.75	RNU2.11P	0.74	GSTA9P	0.61	LOXL2	1.4	TIGD3	0.69	TCTN1	1.5	ZNF771	0.65	CCM2L	1.3	GTSE1.DT	0.69
SFTPB	1.3	AC124944.1	0.71	GSTA4	0.73	PNMA2	1.4	KCNK7	0.76	TMEM116	0.74	PHKG2	0.7	TRPC4AP	1.3	TRMU	0.73
ST3GAL5	1.4	AC069257.3	0.71	AL049555.1	0.74	AC087343.1	0.74	EFEMP2	1.3	TBX5.AS1	1.5	BCL7C	0.74	KIAA1755	1.4	C21orf91	0.67
KRCC1	0.75	RNF168	0.74	SCAT8	0.76	AC124067.2	0.69	RAB1B	1.4	C12orf49	1.4	RAB43P1	1.4	ARHGAP40	1.4	CHODL	0.72
IGKV2.29	1.3	FBXO45	0.74	IBTK	1.6	ZNF703	0.69	TCIRG1	1.4	PXN	1.3	ZNF423	1.3	FAM83D	0.72	LINC01426	0.76
ARID5A	1.3	CEP19	0.74	MRAP2	0.75	ERLIN2	0.75	TPCN2	1.5	NRAV	1.3	ADCY7	1.3	SERINC3	1.4	SETD4	0.72
EIF5B	0.75	BDH1	0.71	NT5E	1.5	PLPBP	0.71	MYEOV	1.4	KMT5A	0.65	NOD2	1.5	PKIG	1.5	CBR1	0.73
IL1RL2	0.71	ZNF718	1.4	SMIM8	0.74	BRF2	0.68	SHANK2	1.4	SNORA9B	0.75	HNRNPA1P48	0.75	STK4	1.3	LINC01679	1.3
LINC01106	0.75	AC079140.2	0.65	RNGTT	1.5	ASH2L	0.72	ALG1L9P	0.73	STX2	1.4	MT1L	1.3	SDC4	1.3	AGPAT3	1.3
IL1B	1.3	SPON2	1.4	GABRR1	0.62												

Stable 4 common genes between DSG and CCM in LUSC

Gene Symbol	Gene ID	P-Value (Survival os)
F10	ENSG00000126218.11	2.34E-03
RXRA	ENSG00000186350.9	3.87E-03
PDE1B	ENSG00000123360.11	3.36E-03
ASL	ENSG00000126522.16	6.35E-03
STXBP1	ENSG00000136854.17	1.12E-02
HAS2	ENSG00000170961.6	8.17E-03
PTEN	ENSG00000171862.9	1.02E-02
FHIT	ENSG00000189283.9	1.08E-02
MDH1	ENSG00000014641.17	3.72E-03
HPRT1	ENSG00000165704.14	5.03E-03
LSM2	ENSG00000204392.10	7.49E-03

Stable 5 common genes between MDSG and CCM in LUSC

Gene Symbol	HR	95% CI	p.value
UPF3B	0.7	(0.53-0.93)	0.013
SEC31A	1.4	(1.1-1.8)	0.018
EIF1AX	0.74	(0.56-0.97)	0.032
CHST7	0.75	(0.57-0.98)	0.037
DGKA	0.74	(0.56-0.98)	0.032
ADCY7	1.3	(1-1.8)	0.04
F10	1.5	(1.1-1.9)	0.0083
STXBP1	1.5	(1.1-1.9)	0.0069
RPS11	0.71	(0.54-0.94)	0.016
ACP1	0.74	(0.56-0.98)	0.035
PHKG2	0.7	(0.53-0.93)	0.014
FBXW5	1.3	(1-1.8)	0.046
AGL	0.75	(0.57-0.99)	0.045
HAS2	1.3	(1-1.8)	0.038
GALT	1.3	(1-1.8)	0.043
GSTA1	0.67	(0.51-0.88)	0.0045
SDC4	1.3	(1-1.7)	0.045
CANT1	1.6	(1.2-2.1)	0.0016
GMPS	0.75	(0.57-0.99)	0.045
EIF4A2	0.72	(0.55-0.96)	0.023
NUP88	0.71	(0.54-0.94)	0.015
MDH1	0.67	(0.51-0.88)	0.0042
DPP4	1.4	(1-1.8)	0.025
INHBA	1.4	(1-1.8)	0.027
EXT1	1.3	(1-1.8)	0.037
SDHD	0.69	(0.52-0.92)	0.01
HIF1A	0.74	(0.56-0.98)	0.036
PTEN	1.4	(1-1.8)	0.027

Stable 6 The selected prognostic genes

Model	Gene Symbol	Coef
1	ASL	0.000829589
1	STXBP1	0.014803016
1	PTEN	0.01134352
1	HPRT1	-0.000736168
2	DGKA	-0.000998893
2	STXBP1	0.004865695
2	ACP1	-0.000864591
2	PHKG2	-0.007937252
2	EIF4A2	-0.000203213
2	HIF1A	-0.000109529
